# Development of a highly effective combination monoclonal antibody therapy against Herpes simplex virus

**DOI:** 10.1186/s12929-024-01045-2

**Published:** 2024-05-28

**Authors:** Narges Seyfizadeh, David Kalbermatter, Thomas Imhof, Moritz Ries, Christian Müller, Leonie Jenner, Elisabeth Blumenschein, Alexandra Yendrzheyevskiy, Frank Grün, Kevin Moog, Daniel Eckert, Ronja Engel, Philipp Diebolder, Mohamed Chami, Jürgen Krauss, Torsten Schaller, Michaela Arndt

**Affiliations:** 1 Heidelberg ImmunoTherapeutics GmbH, Max-Jarecki Str. 21, Heidelberg, 69115 Germany; 2 Biozentrum, University of Basel, Spitalstrasse 41, Basel, CH – 4056 Switzerland; 3 Vanudis GmbH, Max-Jarecki Str. 21, Heidelberg, 69115 Germany; 4 National Center for Tumor Diseases (NCT), Im Neuenheimer Feld 460, Heidelberg, 69120 Germany; 5 Present address: Bio-Rad AbD Serotec GmbH, Anna-Sigmund-Str. 5, Neuried, 82061 Germany; 6 Present address: University of Bern, Institute of Anatomy, Balzerstrasse 2, Bern, 3012 Switzerland

**Keywords:** Herpes simplex virus (HSV), Glycoprotein B (gB), Therapeutic monoclonal antibody, Combination therapy

## Abstract

**Background:**

Infections with Herpes simplex virus (HSV)-1 or -2 usually present as mild chronic recurrent disease, however in rare cases can result in life-threatening conditions with a large spectrum of pathology. Monoclonal antibody therapy has great potential especially to treat infections with virus resistant to standard therapies. HDIT101, a humanized IgG targeting HSV-1/2 gB was previously investigated in phase 2 clinical trials. The aim of this study was to develop a next-generation therapy by combining different antiviral monoclonal antibodies.

**Methods:**

A lymph-node derived phage display library (LYNDAL) was screened against recombinant gB from Herpes simplex virus (HSV) -1 and HDIT102 scFv was selected for its binding characteristics using bio-layer interferometry. HDIT102 was further developed as fully human IgG and tested alone or in combination with HDIT101, a clinically tested humanized anti-HSV IgG, in vitro and in vivo. T-cell stimulating activities by antigen-presenting cells treated with IgG-HSV immune complexes were analyzed using primary human cells. To determine the epitopes, the cryo-EM structures of HDIT101 or HDIT102 Fab bound to HSV-1F as well as HSV-2G gB protein were solved at resolutions < 3.5 Å.

**Results:**

HDIT102 Fab showed strong binding to HSV-1F gB with Kd of 8.95 × 10^–11^ M and to HSV-2G gB with Kd of 3.29 × 10^–11^ M. Neutralization of cell-free virus and inhibition of cell-to-cell spread were comparable between HDIT101 and HDIT102. Both antibodies induced internalization of gB from the cell surface into acidic endosomes by binding distinct epitopes in domain I of gB and compete for binding. CryoEM analyses revealed the ability to form heterogenic immune complexes consisting of two HDIT102 and one HDIT101 Fab bound to one gB trimeric molecule. Both antibodies mediated antibody-dependent phagocytosis by antigen presenting cells which stimulated autologous T-cell activation. In vivo, the combination of HDIT101 and HDIT102 demonstrated synergistic effects on survival and clinical outcome in immunocompetent BALB/cOlaHsd mice.

**Conclusion:**

This biochemical and immunological study showcases the potential of an effective combination therapy with two monoclonal anti-gB IgGs for the treatment of HSV-1/2 induced disease conditions.

**Supplementary Information:**

The online version contains supplementary material available at 10.1186/s12929-024-01045-2.

## Background

Herpes simplex virus types 1 and 2 (HSV-1 and HSV-2) constitute a significant global health concern due to their wide range of clinical manifestations. Infections can affect the skin and mucous membranes, the eyes, the nervous system, or they can lead to disseminated viral spread throughout the body. Clinically, these conditions may manifest as oral and genital herpes, herpes keratitis, herpes encephalitis, and neonatal herpes, respectively [1]. Primary HSV infection occurs through direct contact with a person who is actively shedding the virus, often unknowingly, from their mucosa or skin. The virus persists in nerve ganglia within the dermatome of the primary infection site for a lifetime, potentially causing chronic recurrent mucosal and skin lesions through anterograde axonal transport. HSV-1 typically causes non-sexually transmitted oral herpes infection, while HSV-2 is the most common cause of genital ulcers across the globe [2]. Frequent HSV recurrences often cause significant emotional, psychological and psychosocial distress [3]. Newborns and immunocompromised individuals have a particularly increased risk of morbidity and mortality from HSV infections [4–6]. The significance of developing novel therapies for HSV mediated infectious disease conditions has widely been acknowledged, leading the National Institutes of Health to recently launch a strategic plan aimed at advancing research to improve knowledge on biology and diagnostic possibilities for HSV mediated disease as well as to develop interventions to mitigate its health consequences (nih.gov).

Since many decades virostatic agents (e.g. acyclovir and analogues and derivatives) represent the standard of care treatment of for HSV associated disease. Despite several attempts the development of effective vaccines against HSV has thus far failed [7–9]. Antibody therapeutics have revolutionized treatment options in a vast variety of diseases resulting in the approval of more than 100 products by the US Food and Drug Administration (FDA) [10]. Despite this progress, fewer than 10 monoclonal antibodies (mAbs) targeting pathogens have been FDA approved, and none of those for the treatment of HSV1/2 associated disease conditions. HSV disease animal model studies have shown that UB-621 and HSV8, two fully human IgG1 mAbs, to reduce mortality in an intraperitoneal HSV-1 challenge model of adult mice [11, 12] and to exhibit prophylactic activities in a neonatal mouse infection model [13]. Since topical application of HSV8 protected mice from vaginal transmission of HSV-2 [14], a randomized phase 1 clinical trial of HSV8 in combination with a broadly neutralizing anti-HIV antibody was conducted showing that single and repeated application as a vaginal film (MB66) was safe and well tolerated. Furthermore, an ex vivo bioactivity for both mAbs in vaginal secretions could be demonstrated [15]. A phase 1 dose-escalation clinical trial for subcutaneous administration of UB-621 completed in 2017 proved safety and tolerability in healthy volunteers. In 2023, three phase 2 trials for UB-621 in patients with recurrent genital HSV-2 infection have been started, but are not yet recruiting (NCT03595995, NCT04714060, NCT04979975).

We have developed the humanized monoclonal antibody HDIT101, whose ancestor is the murine monoclonal antibody 2c, which has been generated through hybridoma technology from mice hyperimmunized with HSV-1, binding to glycoprotein B (gB) of HSV-1 and HSV-2, a key component of the cell-entry machinery of HSV. This mechanism allows HDIT101 to neutralize virus particles and effectively inhibit cell-to-cell spread of both HSV-1 and HSV-2. As a result, the antibody has been shown to prevent death in immunodeficient mice challenged with lethal doses of both wild type and multi-resistant HSV strains [16, 17].

After demonstrating excellent tolerability in an intravenous clinical Phase 1 dose escalation trial in healthy volunteers [16], HDIT101 has advanced to a recently completed randomized Phase 2 clinical trial involving 122 patients suffering from chronic recurrent genital herpes. Aim of the trial was to compare the safety and efficacy of a single infusion of HDIT101 with episodic standard-of-care Valaciclovir (VAL) treatment in highly affected patients who have reported at least 4 anogenital HSV-2 recurrences within the last 12 months prior to study enrolment. Based on the highly potent neutralization capacity of HDIT101 in preclinical studies, the ‘percentage of days with lesion recurrences relative to the days on study after initial treatment’ was selected as the primary endpoint of the MATCH-2 trial. While this primary endpoint failed to demonstrate superiority of HDIT101 over standard-of-care treatment improved HDIT101 activity over VAL was observed for the key secondary endpoints ‘mean time to first recurrence’ and ‘mean recurrence rate’*.* Most notably, the delay in mean time to first recurrence in favour of HDIT101 started to become apparent in the Kaplan–Meier plot only from day 35 post infusion, i.e., at a time when the antibody concentration in the circulation was depleted by approximately two half-lives. One possible explanation for the observed long-term effects of HDIT101 could be its role in promoting Antibody Dependent Cellular Phagocytosis (ADCP), involving the uptake of antibody-coated HSV particles by antigen presenting cells, and subsequent T cell activation. Consequently, the observed clinical benefits of HDIT101 in the MATCH-2 trial could likely be the result of the induction of T cell immunity translating into improved recurrence control in chronically infected patients.

Based on these results we were interested to identify more antibodies with similar properties for further potential development into clinical vaccine candidates as either successor of HDIT01 or to be developed in combination with HDIT101. For selection of such antibodies we employed a human antibody phage display library previously been generated from B cell repertoires of tumour draining lymph nodes from head and neck cancer patients, referred to as ‘lymph node-derived antibody libraries’ (LYNDAL) [18]. One of the selected antibodies revealed very similar properties as HDIT01 and was thus formatted into the fully human IgG1 antibody HDIT102 for further characterization.

We here describe the preclinical characterization of HDIT102 and provide a rationale for further developing this antibody as a fully human successor of HDIT101 or as a combinatorial therapeutic. In summary, we have demonstrated in the present study that HDIT102 potently blocked HSV-1 and HSV-2 replication in vitro and in vivo and also exerted synergistical effects with HDIT101 IgG in vivo. We further succeeded in generating cryo-electron microscopic (cryo-EM) co-structures of HSV-1 and HSV-2 gB proteins with HDIT101 and HDIT102 Fabs at resolutions < 3.5 Å so that the exact binding sites of the antibodies could be determined. To our knowledge this is the first report of resolving HSV-2-gB complex. The formation of heterogeneous gB immune complexes observed in the structure may elucidate the synergistic in vivo effects seen in the combination therapy of HDIT101 and HDIT102, as opposed to their monotherapies, presumably by inducing a more robust immune response.

## Material and methods

### Cell lines, viruses and animals

HEK293-6E cell line established from embryonic kidney cells (National Research Council Canada) was used for recombinant antibodies and protein production. HEK293T cell line established from embryonic kidney cells, expressing the SV40 large T antigen (American Type Culture Collection (CRL-3216)) was transiently transfected with gB mutants and was used in fusion and binding assay. Raji cell line established from Burkitt's lymphoma B lymphocytes (American Type Culture Collection) used in ADCC assay. Vero cell line established from normal epithelial kidney cell (American Type Culture Collection) was used for as a permissive cell line for HSV virus in different assays and virus production. Modified Jurkat cells stably expressing the FcγRIIIa receptor, V158 (high affinity) variant, and an NFAT response element driving expression of firefly luciferase provided in ADCC Promega Kit. Clonal HEK293T ectopically and stably expressing HSV-1F gB or gB-GFP were generated by VSV-G pseudotyped lentiviral vector transduction using a transfer vector encoding for codon-optimized HSV-1F gB and subsequent clone selection by limiting dilution. All cell lines were grown in medium recommended by the provider. Primary blood mononuclear cells (PBMCs) were isolated from buffy coats by Ficoll separation and washed in PBS before freezing in freezing media (fetal calf serum (FCS) with 10% dimethyl sulfoxid). Primary CD14 + monocyte-derived macrophages or dendritic cells and CD14- cell fractions containing T-cells were grown in RPMI supplemented with penicillin/streptomycin and 10% fetal calf serum. For all in vivo experiments, 6-week-old female mice (BALB/cOlaHsd) (16–19 g) were purchased from ENVIGO and housed according to regulatory guidelines.

### Virus propagation and titration

HSV-1F and HSV-2G virus stocks were purchased from ATCC and expanded in Vero and used for in vitro and in vivo experiments. Vero cells were cultured until reaching 100% confluency in T175 flasks. The culture medium was removed and after one wash with PBS infected with HSV-1F/HSV-2G at MOI of 0.01 in DMEM without FCS for 2h at 37°C. 30mL medium with 10% heat inactivated FCS was added and the infected cells were incubated 2–3 days. The flasks were subjected to three freeze–thaw cycles (-80°C, room temperature (RT)), to lyse the cells and release virus into the supernatant. Cell debris was removed by centrifugation (15min, 300g) and the virus-containing supernatant filtered using a 0.45μm filter, followed by ultracentrifugation at 20k rpm for 1 h (SW32Ti swinging rotor, Beckman Coulter). The virus pellets from nine infected T175 flasks were pooled in 1 mL PBS and aliquots were stored at -80°C. The titers of HSV stocks were determined by TCID50 assay (tissue culture infectious dose 50%). For this purpose, 1 × 10^4^ Vero cells were plated per well in 100μl in a 96-well plate. The following day, a 1:10 dilution series of virus, starting with 1:1000 dilution of stock was prepared. The medium in the 96-well plate was replaced by 100 μL virus dilution per well, for each dilution step 10 wells were used. After 2 h incubation at 37°C, supernatant was replaced with fresh DMEM supplemented with 10% heat inactivated FCS and the plates were incubated at 37°C. After 3 days, the plates were examined under the light microscope for cytopathic effect and the TCID50/ml of virus stock was calculated.

### Selection, cloning, expression, and purification of IgG

To select human Herpes Simplex Virus Type 1 (HSV-1) specific single chain fragment variables (scFvs), a human lymph node-derived antibody phage display library was screened against recombinantly expressed glycoprotein B ectodomain from HSV-1 (strain KOS) [18]. According to the binding profile and HSV neutralization capacity, the most promising candidate, HDIT102, was chosen for further investigation. To generate an IgG with the same constant domains as HDIT101, codon-optimized variable heavy chain (VH) and variable light chain (VL) cDNAs of the selected scFv were synthesized appropriate restriction sites for cloning into pConPlus vectors containing IgG1 heavy/light-chain constant regions cDNAs. The synthesized VH coding sequences were cloned into pConPlus containing heavy chain constant region using *Hind*III and *Apa*I. VL coding cDNA was cloned into pConPlus Lambda encoding light-chain constant region using *Hind*III and *Avr*II. Afterwards, pConPlus vectors encoding light chain and heavy chains were cut with *Not*I and *Pvu*I and ligated to generate a double-gene vector. HDIT101 was produced at GMP-grade and HDIT102 at research grade quality by contract manufacturers.

### HSV antibody neutralization assay

An end point titration assay was used to assess the antiviral activity of HDIT101, HDIT102, and a combination of both antibodies against cell-free virus. Briefly, different antibody dilutions were incubated with a constant viral dose (100 TCID50 HSV-1F or HSV-2G) for 1 h at 37°C. The antibody-virus mixtures were applied to 80–90% confluent Vero cells in 96-well plates (2.0 × 10^4^ cells per well) in a volume of 100μL per well. As a control, Vero cells were infected with a viral dose of 100 TCID50 without prior incubation with antibody. The extent of the cytopathic effect was examined by light microscopy three days after infection. The neutralization titre was determined to be the highest antibody dilution at which the virus was completely neutralized and the formation of a CPE in the inoculated cell cultures was completely prevented. In addition, the neutralizing antibody concentration at which 50% of the cell culture wells are protected from infection (IC50) were calculated as described before [19].

### Cell to cell spread inhibition assay

2.0 × 10^5^ Vero cells were seeded in each well of a 4-well chamber slide. The following day, the culture medium was discarded, and the cells were inoculated with a viral load of 200 TCID50 HSV-1F or HSV-2G in a volume of 500 μL DMEM per well. Four hours after infection, the supernatant containing virus was discarded, unbound viral particles were removed by washing one time with 500 μL PBS, and the cells were incubated with 500 μL culture medium containing 10% FCS and 500 nM of HDIT102. As a control for inhibition of cell to cell spread, human polyclonal anti-HSV antibodies (Enzygnost) was used with a dilution of 1:20 in culture medium. After two days of incubation, the medium was discarded. The cells were washed once with PBS and then fixed with 5% paraformaldehyde solution and washed again with PBS. Subsequently, HSV-infected cells were stained using a FITC-conjugated anti-HSV antibody. After one hour, the supernatant was discarded, and cells were washed with PBS to remove excess antibodies. Afterwards, the cells were subjected to staining with Hoechst to visualise nuclei and then fixed with 5% paraformaldehyde for 15 min. The evaluation was carried out by 20 × magnification fluorescence microscopy using an inverted microscope (Leica).

### Mouse HSV infection model

In vivo efficacy of the HDIT102 or Fc mutant N297A was investigated in an HSV-2 infection BALB/cOlaHsd mouse model. One week prior to the experiment, 6-week-old female mice BALB/c (weight: 16–19 g) were purchased from ENVIGO and one week prior to virus inoculation pre-treated subcutaneously with medroxyprogesteron (longacting progestin Depo-Clinovir, prepared at 25 mg/mL in PBS and 100 μL per mouse). On the day of intravaginal virus inoculation, the experimental animals were anesthetized by isoflurane. During the short anaesthesia, the vaginal mucosa was cleaned from the vaginal secretions by using a sterile ESwab and the experimental animals were infected by intravaginal inoculation of 10 μL of virus stock (containing 5.0 × 10^4^ TCID50 HSV-2G) to the vaginal mucosa using a pipette. Afterwards, a small amount of Epiglu tissue adhesive was applied on the surface to temporarily glue the vagina (avoids inoculum to flow out). The glue was lost within 1 day after inoculation. The efficiency of antibodies in protecting mice from a lethal HSV-2G infection was assessed by the intraperitoneal administration after infection. The experimental animals were regularly inspected for weight loss and the occurrence of perineal hair loss (HL), redness (R) and swelling (S) and neurologic damage (e.g. hind limb paralysis, gastrointestinal track blockage) over an observation period of 60 days. Visible inspection was graded from slight to severe symptoms accordingly + / +  + / +  +  + . Experimental animals were sacrificed in case of severe signs of herpes encephalitis, or paralysis or occurrence of severe lesions to prevent undue suffering. All experiments were done in line with ethical approval. To demonstrate antiviral activity doses of 300 μg or 600 μg HDIT102 (each *n* = 10) and 600 μg HDIT102-N297A (*n* = 5) in 100 μL PBS were tested. Control groups were treated with PBS. For combination experiments HDIT102 IgG was pre-mixed with HDIT101 IgG at equimolar ratio (combination therapy) and injected at a final total IgG dose of 300 μg intraperitoneally. HDIT101 or HDIT102 alone (monotherapy) were injected intraperitoneally at the same dose. 20 mice per treatment group and 15 mice for the control arm were used in total. The statistical differences between survival curves were calculated using Logrank Mantel Cox test. The differences between cumulative combined clinical scores were analyzed using Kolmogorov-Smirnow test.

### Competition binding assay

Competitive binding assays with HDIT101 and HDIT102 were done by flow cytometry and enzyme-linked immunosorbent assay (ELISA). HEK293T cells expressing HSV-1F gB were incubated with 10 μg/mL of the murine ancestor of HDIT101 (MAb2c) in combination with a serial dilution of either the humanized IgG HDIT101, the fully human IgG HDIT102, or a none competing control IgG (huRFB4). Subsequently, the cells were stained with anti-murine IgG-APC and analyzed in flow cytometry. For ELISA His-tagged Fab102 was added in twofold serial dilutions to either HSV-1 gB or HSV-2 gB coated microtiter plates. After 1.5 h at room temperature wells were washed and subsequently incubated for 1 h with Penta His-HRP conjugate (1:20,000). Wells were washed again prior to incubation with a 100-fold molar excess Fab101 or blocking buffer for 1.5 h. After a final wash, bound Fab102 was detected using TMB chromogenic substrate and signal was detected using a Tecan plate reader.

### Cross-reactivity binding assay to other herpesviridae members

To check for HDIT102 cross-reactivity with other common members of the herpesviridae family, commercial Enzygnost anti-HSV/VZV/CMV/EBV IgG kits were used according to the manufacturer’s instructions. Bound antibodies were detected with rabbit anti-human Fcγ IgG-HRP conjugated polyclonal antibody (Jackson ImmunoResearch).

### ADCC assay

The test antibodies were incubated with HSV-infected Vero cells or HEK293T cells stably expressing gB and engineered Jurkat reporter cells stably expressing the FcγRIIIa receptor, V158 (high affinity) variant, and an NFAT response element driving expression of firefly luciferase (Promega). Vero cells were infected with HSV-1F/2G at MOI 1. Twenty hours after infection, cells were harvested and distributed in white flat bottom 96 well plates (1.25 × 10^4^ cells per well) and incubated 6 h together with Jurkat effector cells at an effector:target ratio of 6:1 and serial dilutions of test antibodies. Noninfected Vero cells and Raji cells incubated with Rituximab served as negative and positive controls, respectively. Luciferase substrate was added and after 15 min incubation luminescence intensity was measured using a plate reader. Triplicate reads were performed, and means were calculated. Plate Background from control wells was subtracted. Fold of ADCC induction was calculated.

### CDC assay

To measure and compare complement activation in the presence or absence of neutralizing antibodies, Vero cells were infected with HSV-1F or HSV-2G at an MOI of 1 for 20 h in T75 flasks (containing 4 × 10^5^ cells). After the incubation time the infected Vero cells were harvested and washed once with PBS. Afterwards, 75 μg of neutralizing antibodies were prepared in DMEM with 20% heat-inactivated or not heat-inactivated human IgG-depleted serum and added to 5 × 10^5^ infected Vero cells and incubation was carried out for 4 h at 37°C. Then, the supernatant was subjected for the quantitative determination of complement activity using a commercial ELISA kit designed to measure human terminal complement complex C5b-9 (TCC C5b-9) concentration by following the protocol of the kit (Human terminal complement complex C5b-9 ELISA, Creative Biolabs). The assay is based on a sandwich enzyme immunoassay technique. In addition, infected Vero cells were washed with PBS once and stained with SYTOX Blue (Dead Cell Stain) for 15 Minutes at RT to discriminate dead and viable cells. subjected.

### Alanine scanning mutagenesis and fusion inhibition assay

Specific HSV-1F gB amino acid residues in close proximity to HDIT101 or HDIT102 CDR residues in the cryo-EM co-structure were interrogated for their contribution to IgG-mediated inhibition of gB-induced cell–cell fusion by substitution to alanine. Mammalian expression plasmids encoding wild type HSV-1F gB or single amino acid point mutants were co-transfected into a 1:1 mix of HEK293T-GFP and HEK293T-E2C fluorescent reporter cells, together with mammalian expression plasmids encoding HSV-1F gD, gH and gL protein. Transfected cells were incubated at 37°C and 5% CO_2_ for 5 h before HDIT101 or HDIT102 was added to final concentration of 75μg/mL. In control samples no antibody was added. Fusion of cells was judged by the presence of GFP + E2C + double positive cells by flow cytometry two days later.

### Generation of HDIT101/HDIT102-resistant mutant viruses in vitro

Vero cells (8 × 10^6^ cells in T75 flask) were infected with HSV-1/2 at MOI 0.01 and passaged in the presence of increasing concentrations of neutralizing mAbs in multiple rounds. After each round, viral supernatant was harvested, purified through 0.45 μm filter before inoculating fresh Vero cells. After 4 rounds of passaging, harvested virus was characterized for its neutralizing potency in the absence or presence of neutralizing antibody concentrations and an aliquot of infected cells was used for DNA extraction (QiaAmp, Qiagen). Partial gB coding regions were amplified by PCR and products were Sanger-sequenced (Eurofins MWG) to determine resistance mutations. Clonal resistant viruses were grown by limiting dilution of resistant virus pool and expansion of single plaques under antibody pressure.

### CD14 + monocyte selection and macrophage/dendritic cell differentiation and phagocytosis assay

Monocyte-derived macrophages (MDMs) were generated from buffy-coat primary blood mononuclear cells (PBMCs) of three independent healthy donors by isolation of CD14 + monocytes by magnetic separation using MACS microbeads (Miltenyi) and differentiation using 80 ng/mL granulocyte–macrophage colony stimulating factor (GM-CSF) to generate type 1 MDMs or 50 ng/mL M-CSF to generate type 2 MDMs or 80 ng/mL GM-CSF + 20 ng/mL interleukin 4 to generate monocyte-derived dendritic cells (MDDC) for 7 days. The differentiated cells were then exposed to HSV-1 labelled with a pH-sensitive dye (IncuCyte pHrodo Orange Cell Labeling Dye, Sartorius) at an MOI of 10 in the presence or absence of 150 μg/mL HDIT101 and/or HDIT102 antibody or 50 μg/mL Acyclovir. Three technical replicates were then monitored using an Incucyte system (Sartorius) at intervals of one hour. The amount of virus taken up as measured by dye fluorescence was normalized to the cell count in the imaged area.

### Macrophage-dependent activation of T cells

Autologous T-cell activation was measured after exposure of HSV-HDIT101 and/or HDIT102 immune complexes to MDMs from two independent HSV-seropositive healthy donors. MDMs were pre-incubated for 24 h with HSV-1F (MOI 10) in the presence or absence of neutralizing amounts of HDIT102 and/or HDIT101 before addition of autologous T-cells. Analysis was done by flow cytometry using BV785-labeled anti-CD69 IgG (Biolegend) as T-cell activation marker, APC-labelled anti-CD4 (Biolegend) and BV605-labeled anti-CD8 (Biolegend) 24 h after addition of the autologous CD14- fraction to the pre-incubated MDMs. Percentage of CD69-positive cells as well as mean fluorescent intensity (CD69 MFI) were measured and analyzed separately for CD4 + as well as CD8 + cells.

### Protein production

GMP-grade HDIT101 IgG1 was produced for clinical trials by Celonic Germany GmbH. The Fab proteins were prepared by Papain digestion of HDIT101. For this, we used a Pierce Fab Preparation Kit (ThermoFisher) and dialyzed the obtained protein 3 times against 50 mM Tris, 150 mM NaCl, pH 8 buffer. HDIT102 Fab was generated recombinantly by transient expression in HEK293-E6 cells. For this the HDIT102 VH-CH1 domain fused to a double Strep-tag and the HDIT102 light chain were cloned into a mammalian expression vector.

The codon-optimized sequence of HSV-1F gB (aa 30–729; UniProtKB P06436.1) and HSV-2G gB (aa 22–724; UniProtKB: A0A410TI43) ectodomain proteins including a BM40 signal peptide and a C-terminal double Strep-tag were cloned into a mammalian expression vector. The proteins were then transiently expressed in HEK293-E6 suspension cells cultured in F17 medium (ThermoFisher) supplemented with 0.1% Kolliphor (Sigma) and 4 mM Glutamine. HEK293 cells were transfected with PeiMax (Polysciences) at a cell density of 1.5–2 × 10^6^ cells/mL with 1 μg plasmid and 2 μg PeiMax/mL culture media. 24 h after the transfection Tryptone N1 feeder (Organi Technie) was added to the cultures. On day 5 after the transfection the supernatant was harvested by two centrifugation steps, first 1200 rpm to remove the cells and then 3600 rpm to remove cell debris. Next, the pH of the supernatant was adjusted by the addition of 1 mL 2 M Tris buffer pH 9 / 100 mL supernatant. We then purified both proteins by Strep-Tactin XT (IBA, Germany) gravity flow purification according to the manufacturer protocol.

The Strep-tag of both gB proteins was then removed by thrombin digestion (Serva) and dialysis against 50 mM Tris, 150 mM NaCl, pH 8. The thrombin digested gB proteins were then purified 3 × times by Strep-Tactin XT affinity chromatography to deplete the sample from any remaining Strep-tagged protein. Next, we concentrated the proteins with Amicon spin columns (cut-off 30kD) and the proteins were further purified with a Superdex 200 10/300 GL SEC column and an Äkta Pure FPLC system. The peek fractions were pooled and concentrated with Amicon spin columns.

### Determining co-structures by cryo-EM

For cryo-EM grid preparation and data collection, the following steps exemplified for HSV-2 gB and HDIT101 Fab were performed. Procedures to solve cryo-EM structures HSV-2 gB/HDIT102 Fab, HSV-1 gB/HDIT101 Fab and HSV-1 gB/HDIT102 Fab were similar, and details are shown in Tables 1 and 2, respectively. The procedure for one of the complexes is described in more detail here: Recombinant HSV-2G gB ectodomain and HDIT101 Fab were mixed in a ratio of 1 to 3.5. A 4 μl aliquot of the mixture was adsorbed onto glow-discharged Quantifoil Au-R2/1-300mesh holey carbon-coated grids (Quantifoil, Germany), blotted with Whatman 1 filter paper and vitrified into liquid ethane at -180°C using a Leica EM GP2 plunger (Leica microsystems, Austria) operated at 10°C and 85% humidity. Data was acquired on a Glacios TEM (ThermoFisher) operated at 200 kV and equipped with a Quantum K3 direct electron detector (Gatan). Micrograph movies of 40 frames were recorded in counting mode at a magnification of 45,000 × (pixel size 0.878 Å) with a dose of 1.325 e^−^/Å^2^/frame, resulting in a total accumulated dose on the specimen level of approximately 53 e^−^/Å^2^ per exposure. All image processing steps were performed with Relion v4.0 [20]. Dose weighting and motion correction of dose-fractionated and gain-corrected movies were performed using Relion’s implementation of the UCSF motioncor2 program. Contrast transfer function (CTF) parameters were estimated using ctffind 4.1.14 [21]. Micrographs displaying strong drift, astigmatism greater than 1000 Å and maximum CTF resolution worse than 8 Å were excluded from further processing. A total of 6 million particles were picked using the Laplacian-of-Gaussian (LoG) filter in Relion 4.0 [20]. The particle dataset was cleaned through five rounds of reference-free 2D classification resulting in 915′372 particles. Relion’s Stochastic Gradient Desecnt (SGD) algorithm was used to generate a de novo 3D initial model from the 2D particles. The particle dataset was further cleaned through three rounds of unsupervised 3D classification. The remaining 234′096 particles were subjected to Bayesian particle polishing, CTF and aberration refinement, and a final high-resolution 3D refinement, which resulted in a final map with an overall resolution of 3.45 Å according to the gold standard Fourier shell correlation (FSC) at FSC = 0.143 (Fig. S3). The HSV-1 gB X-ray structure (PDB-ID: 2GUM) was manually mutated according to a sequence alignment with sequence QAU10436.1 (UniProt entry A0A410TI43) and placed into the final map using coot [22]. For the HDIT101 Fab, the crystal structure of a humanized recombinant Fab fragment of a murine antibody (PDB-ID 3AAZ) was mutated in coot [22] based on a sequence alignment generated by Needle EMBOSS [23]. Three HDIT101 Fabs were placed into the final map using coot [22]. Molrep of the CCP-EM software suite v1.6 [24] was used for the initial fitting of gB and the three HDIT101 Fabs into the final map. The final protein model was obtained by several iterations of manual model building in coot [22], Refmac-Servalcat refinement and model validation in the CCP-EM software suite v1.6 [24].

**Table 1:** Cryo-EM data collection, refinement and validation statistics

**Data collection**	**HSV-1F gB/HDIT101 Fab**	**HSV-2G gB/HDIT101 Fab**
Microscope	Glacios	Glacios
Voltage (kV)	200	200
Magnification	45′000	45′000
Pixel size (Å)	0.878	0.878
Collection software	Serial EM	Serial EM
Defocus range (μm)	-1.0 to -2.0	-1.0 to -2.0
Movies recorded	5′815	9′700
Frames per movie	40	40
Exposure rate (e^−^/Å^2^/frame)	1.25	1.325
Exposure time (s)	4	3.2
Cumulative exposure (e^−^/Å^2^)	50	53
**Refinement**		
Software	Relion v4.0	Relion v4.0
Initial particle images (no.)	2′993′934	6′289′845
Final particle images (no.)	233′330	234′096
Symmetry imposed	C_1_	C_1_
0.143 FSC half map (Å)	3.27	3.45
**Map sharpening B-factor (Å**^**2**^**)**	-50.38	-79.24
**Model building and validation**		
Software	CCP-EM v1.6 coot v0.9.8	CCP-EM v1.6 coot v0.9.8
MolProbity score	1.71	1.63
All-atom clashscore	12.28	10.74
Rotamer		
Favored (%)	97.98	98.08
Outliers (%)	0.19	0
Ramachandran		
Favored (%)	97.44	97.64
Outliers (%)	0	0
CaBLAM outliers (%)	1.09	1.1
Cβ outliers (%)	0	0
FSC 0.5 average (%)	78.62	79.99

**Table 2:** Cryo-EM data collection, refinement and validation statistics

**Data collection**	**HSV-1F gB/HDIT102 Fab**	**HSV-2G gB/HDIT102 Fab**	
Microscope	Titan Krios G1	Glacios	
Voltage (kV)	300	200	
Magnification	165′000	45′000	
Pixel size (Å)	0.82	0.878	
Collection software	Serial EM	Serial EM	
Defocus range (μm)	-1.0 to -2.0	-1.0 to -2.0	
Movies recorded	6′611	17′039	
Frames per movie	40	40	
Exposure rate (e^−^/Å^2^/frame)	1.15	1.256	
Exposure time (s)	4	3	
Cumulative exposure (e^−^/Å^2^)	46	50	
**Refinement**			
Software	Relion v4.0	Relion v4.0	
Initial particle images (no.)	1′059′496	10′263′500	
Final particle images (no.)	208′280	575′340	
Symmetry imposed	C_1_	C_1_	
0.143 FSC half map (Å)	3.44	3.12	
Map sharpening B-factor (Å^2^)	-74.05	-80.47	
**Model building and validation**			
Software	CCP-EM v1.6 coot v0.9.8	CCP-EM v1.6 coot v0.9.8	
MolProbity score	1.37	1.39	
All-atom clashscore	6.77	7.05	
Rotamer			
Favored (%)	98.92	99.27	
Outliers (%)	0	0	
Ramachandran			
Favored (%)	97.68	98.08	
Outliers (%)	0	0	
CaBLAM outliers (%)	1.30	1.20	
Cβ outliers (%)	0.10	0	
FSC 0.5 average (%)	80.11	83.6	

### Bio-layer interferometry (BLI)

First, HSV-1/2 gB ectodomain proteins were dialyzed 3 times against PBS buffer, then the proteins were biotinylated with EZ-Link NHS-PEG4-Biotin reagent (ThermoFisher) at a molar ratio of gB protein as monomer to biotin equals 1:3. Next, the proteins were dialyzed to PBS buffer once and stored at 4°C. For BLI measurements we used a Sartorius Octet R8 machine, Octet Streptavidin biosensors and Octet BLI Discovery 12.2.2.20 software. In the beginning we performed ligand loading optimization experiments with different ligand concentrations and a fixed analyte concentration. All samples were diluted in kinetic buffer (PBS pH 7.4 with 0.02% Tween-20, 0.1% albumin (Sigma) and 0.05% sodium azide).

For the kinetic measurement we used the determined optimal ligand concentration of HSV-1/2 biotinylated gB protein and serial dilutions of the analyte HDIT101 or HDIT102 IgG or Fab. All the kinetic measurements were then performed in triplicate. The binding data were then analysed with Octet Analysis Studio 12.2.2.26 software (Sartorius).

### Positive selection analysis

HSV-1 and HSV-2 gB DNA sequences were retrieved by nucleotide BLAST (NCBI) against HSV-1F gB or HSV-2G gB sequences. Sequences were aligned using DNADynamo (Bluetractor software). Only full lengths sequences without any non-assignable nucleotides or frame shifts were considered in the alignment. In total 451 HSV-1 gB and 368 HSV-2 gB cleaned DNA sequences were retrieved. Positive selection analysis was performed using single-likelihood ancestor counting (SLAC) (www.datamonkey.org) for 185 non-identical HSV-1 and 201 non-identical HSV-2 gB sequences [25]. Phylogenetic trees were visualized using FigTree.

## Results

### Binding characteristics of the fully human IgG HDIT102

**Fig. 1:**
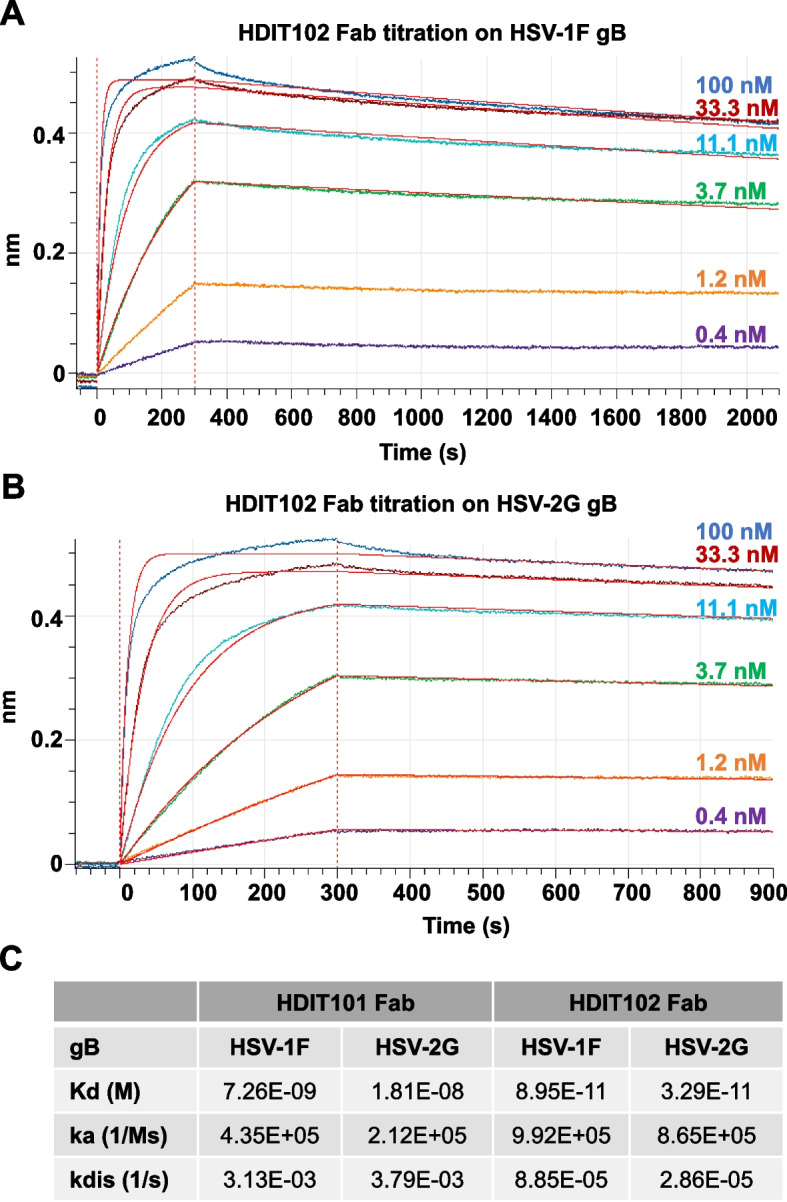
Bio-layer interferometry (BLI) analysis of recombinant HSV-1 or HSV-2 gB interaction with HDIT101 or HDIT102 Fab. Biotinylated gB of HSV-1F (**A**) or HSV-2G (**B**) was immobilized on streptavidin biosensor tips and incubated with a serial dilution of HDIT102 (100, 33.3, 11.1, 3.7, 1.24 or 0.41 nM). Octet sensorgrams were recorded. **C** Measurements of association (ka) and dissociation (kdis) rates and calculation of binding affinities using a dilution series of HDIT101 or HDIT102 Fab binding to recombinant HSV-1F or HSV-2G gB

Glycoprotein B plays a crucial role in the viral entry process and is indispensable for HSV replication and pathogenesis. We generated HDIT102, a fully human IgG1 antibody, whose variable domains were isolated from an scFv phage display library utilizing HSV-experienced B cell repertoires, through targeted selection against gB of HSV-1. Glycoprotein B is a highly conserved viral protein found among herpesviruses. To exclude potential cross-reactivity of HDIT102 to other members of the herpes virus family, it was demonstrated that the IgG binds specifically to HSV and not to VZV, HCMV and EBV using routine immunodiagnostics ELISAs for virus specific serology (Fig. S1A). We then compared the binding affinity of HDIT102 with that of the antibody HDIT101, a humanized IgG1 currently in clinical development, using biolayer-interferometry (Octet, Sartorius). The association rates (ka) for HDIT101 and HDIT102 IgG were comparable (5.0 × 10^5^ and 7.0 × 10^5^ M^−1^s^−1^). However, in contrast to HDIT101, HDIT102 IgG exhibited an exceptionally slow dissociation rate (kdis), rendering it non measurable (Fig. S1B, C). Due to the absence of measurable kdis for HDIT102, the Kd binding constant for its IgG format could not be determined. Therefore, Fab fragments of both antibodies, Fab101 and Fab102, were generated for investigating the monovalent binding kinetics. Fab102 had slightly increased association rates (ka) to HSV-1F gB (9.92 × 10^5^ M^−1^s^−1^) as well as to HSV-2G gB (8.65 × 10^5^ M^−1^s^−1^) as compared to Fab101 (HSV-1F gB, 4.35 × 10^5^ M^−1^s^−1^ and HSV-2G gB, 2.12 × 10^5^ M^−1^s^−1^) (Fig. 1A, B and C). The dissociation rate for Fab102 was dramatically decreased compared to Fab101 for both HSV-1F (Fab102 vs. Fab101, 8.85 × 10^–5^ s^−1^ vs. 3.13 × 10^–3^ s^−1^) and HSV-2G (Fab102 vs. Fab101, 2.86 × 10^–5^ s^−1^ vs. 3.79 × 10^–3^ s^−1^), mirroring the pattern observed with HDIT102 IgG. This leads to a significantly lower Kd for Fab102 when binding to HSV-1F gB (Fab102 vs. Fab101, 8.95 × 10^–11^ M vs. 7.26 × 10^–9^ M) and HSV-2G gB (Fab102 vs. Fab101, 3.29 × 10^–11^ M vs. 1.81 × 10^–8^ M).

### HDIT102 exhibits potent neutralization capacity in vitro and prevents disease in vivo

**Fig. 2:**
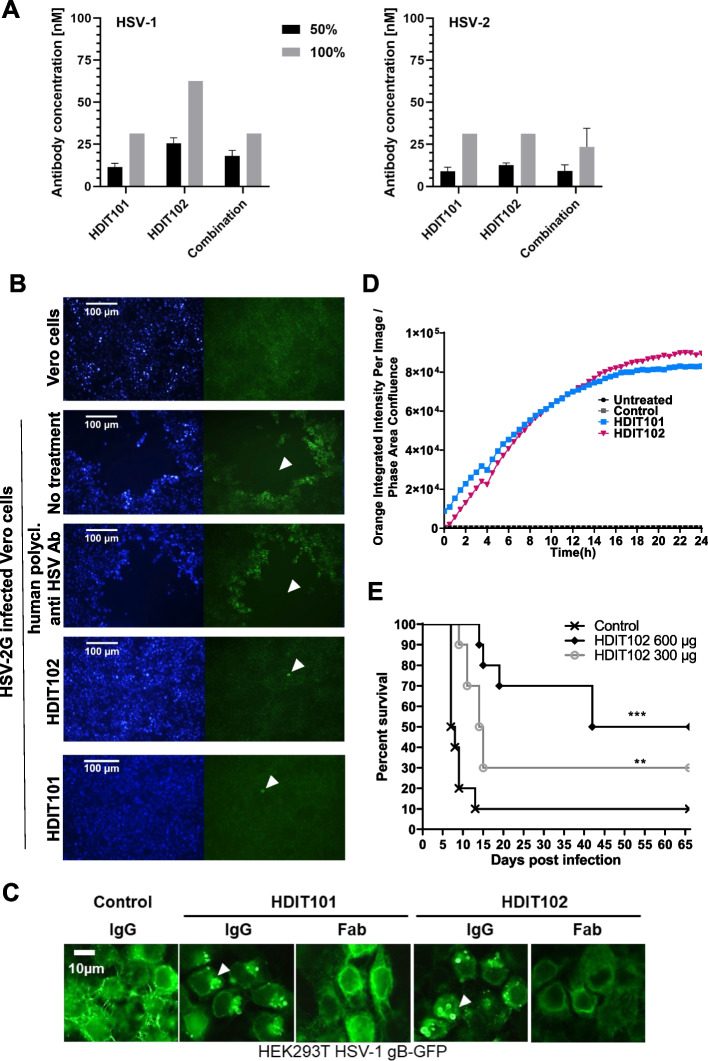
In vitro antiviral activities and in vivo efficacy of HDIT102. **A** The antibody concentration required for reducing the virus-induced cytopathic effect (CPE) by 50% and 100% was determined by an endpoint dilution assay. Serial dilutions of antibodies were incubated with 100 TCID50 of HSV-1F or HSV-2G. The antibody virus inoculum was applied to Vero cell monolayers grown in microtiter plates and CPE was scored after 72 h. Means and error bars, showing standard deviation of mean, were calculated based on three independent experiments. **B** Inhibition of HSV-2G cell-to-cell spread by HDIT101 and HDIT102. Fluorescence microscopy images of Vero cells infected with HSV-2G and subsequently treated with either HDIT101 (75 μg/ml), HDIT102 (75 μg/ml), human polyclonal anti-HSV antibody (1:20) or left untreated. Plaque formation was visualised by anti-HSV immuno- and Hoechst staining. Representative images are shown. Arrows show plaques or initially infected cells. **C** Fluorescence microscopy images of HEK293T cells ectopically expressing HSV-1 gB-GFP and treated with either 5 μg/mL HDIT101 or HDIT102 (IgG vs. Fab) or with an irrelevant isotype control antibody. **D** Incucyte data of HSV-1F-infected Vero cells incubated with either HDIT101, HDIT102 or isotype control IgG labelled with a pH-sensitive dye. Uptake of IgGs into the endosomal pathway was measured over time by tracking fluorescence. **E** HDIT102 treatment of immunocompetent BALB/cOlaHsd mice after a lethal intravaginal HSV-2G infection. The mice were infected with HSV-2G (5 × 10^4^ TCID50) intravaginally and four hours later 600 μg or 300 μg of HDIT102 were injected intraperitoneally, while the control group received PBS (each group, *n* = 10). The statistical differences between survival curves were calculated using Logrank Mantel Cox test, ** *p* < 0.05, *** *p* < 0.001

To examine the antiviral activity of HDIT102 towards cell-free virus, neutralization capacities of different antibody dilutions were determined for HSV-1F or HSV-2G. As control, Vero cells were infected without prior incubation of virus with antibody. The highest antibody concentration preventing the viral cytopathic effect (CPE) to 50% and 100% relative to the control were determined three days after infection and considered the endpoint. Although HDIT102 exhibited a significantly higher affinity than HDIT101, its in vitro neutralization capacity for HSV-2 was comparable to that of HDIT101, whereas for HSV-1, concentrations twice as high as those of HDIT101 were required for 50% and 100% neutralization. Inhibitory concentrations for neutralizing HSV-1F by HDIT102 were 25.5 nM and 62.5 nM, while inhibitory concentrations for HSV-2G were 12.5 nM and 31.25 nM, for 50% and 100% neutralization, respectively (Fig. 2). Furthermore, the combination of both antibodies in a 1:1 ratio maintained neutralization efficiency comparable to that of a single antibody treatment. The combination prevented virus-induced CPE of HSV-1 by 50% at a concentration of 18 nM and by 100% at 31.25 nM. For HSV-2, the concentrations required were 9.3 nM to achieve 50% and 23.44 nM for 100% neutralization (Fig. 2).

Cell-to-cell spread has been suggested as the predominant way of transmission in vivo and titres of antibodies preventing cell-to-cell spread have been proposed to correlate with reduced number of recurrences for HSV-1 [26]. HDIT101 has been demonstrated to block cell-to-cell transmission in Vero cells [17]. Similarly, HDIT102 blocked cell-to-cell transmission of HSV-1F or HSV-2G in Vero cells (Fig. 2B and Fig. S2B). We speculated that both HDIT101 and HDIT102 may block cell-to-cell spread by binding to gB on the cell surface before gB becomes internalized and incorporated into membranes of newly produced viruses. When HEK293T cells expressing HSV-1 gB carboxyterminally fused to GFP were treated with HDIT101 or HDIT102, large aggregates of gB-GFP could be detected that were dependent on the presence of bivalently binding IgGs since aggregates were absent for monovalently binding Fab fragments (Fig. 2C). To demonstrate this for gB expressed on infected cells, Vero cells infected with HSV-1 were treated with either HDIT101 or HDIT102 IgG being labelled with a pH-sensitive dye and uptake into endosomes was measured over time. HDIT101 as well as HDIT102 induced endosomal internalization of gB in infected Vero cells, suggesting that cell-to-cell spread is blocked by IgG recruitment of cell-surface exposed gB to endosomes trapping and inactivating progeny viruses before or at the stage of gB incorporation into the viral membrane (Fig. 2D). To demonstrate antiviral activity of HDIT102 in vivo, immunocompetent BALB/c mice were intravaginally infected with a lethal dose of HSV-2G and treated afterwards with 300μg or 600μg HDIT102 intraperitoneally. Significantly more mice survived in the HDIT102 treatment groups as compared to the control group and a dose-dependent effect was observed (Fig. 2E).

### HDIT101 and HDIT102 bind to overlapping epitopes in gB domain I and can form heterogenic immune complexes with trimeric gB

**Fig. 3:**
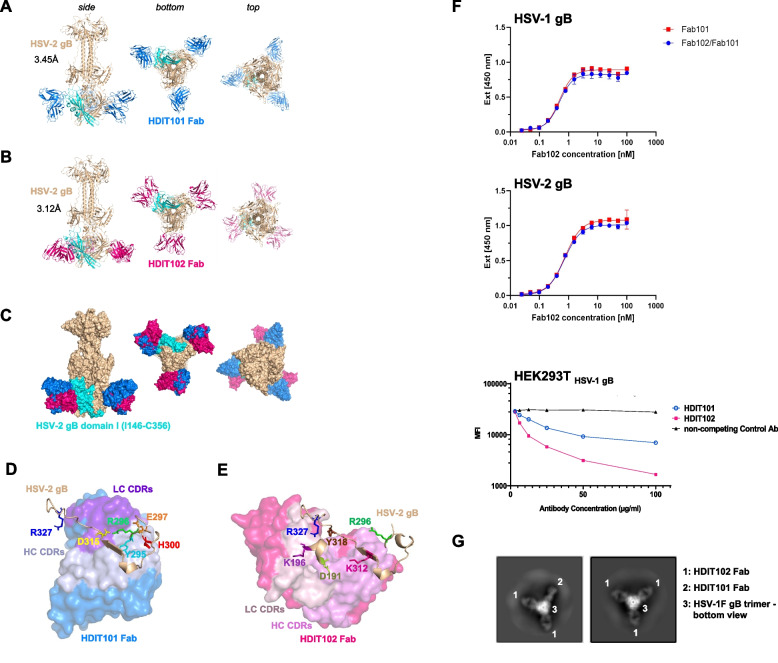
Determination of the cryo-EM structure of HDIT101 or HDIT102 Fab bound to trimeric HSV-2G gB at a resolution < 3.5 Å. **A** Side, bottom and top view of co-structure of trimeric HSV-2G gB in post-fusion conformation (brown) with three HDIT101 Fab molecules (blue) solved by cryo-EM at a resolution of 3.45 Å are shown in cartoon representation. HSV-2 gBG domain I (amino acid residues I146-C356) is highlighted in cyan. **B** The co-structure of HDIT102 Fab (magenta) bound to trimeric HSV-2G gB in post-fusion conformation (brown) was determined at a resolution of 3.12 Å and side, bottom and top views are shown in cartoon representation. **C** Overlay of the co-structures represented as surface models indicating overlapping epitopes of HDIT101 and HDIT102 Fabs on HSV-2G gB indicating perpendicular orientation of both Fabs to another. **D** Critical HSV-2G gB residues in close contact with HDIT101 HC (light blue) and LC (violet) CDR residues are are shown as sticks and highlighted in different colours. **E** Critical HSV-2G gB residues in close contact with HDIT102 HC (pink) and LC (light pink) CDR residues are shown as sticks and highlighted in different colours. **F** Competitive binding towards HSV-1 gB and HSV-2 gB of HDIT101 and HDIT102 was performed by ELISA using their respective Fab fragments. Binding of Fab102-His at increasing concentrations was detected with a Penta His-HRP conjugate and chromogenic substrate TMB in the presence or absence of Fab101 at 100-fold molar excess. Competition assay of the humanized IgG HDIT101 and human IgG HDIT102 to clonal HEK293T cells ectopically expressing HSV-1 gB. HEK293T HSV-1gB cells were incubated with a fixed amount of fluorochrome-labelled murine MAb2c IgG2 containing identical CDRs to HDIT101 in combination with increasing amounts of either HDIT101, HDIT102 or an irrelevant human IgG (anti-CD22). Mean fluorescent intensities (MFI) were measured by flow cytometry. **G** 2D-class averages from cryo-EM analyses of recombinant trimeric HSV-1F gB mixed with 1:1 molar ratio of HDIT101 and HDIT102 Fabs. gB trimers with heterogenic binding of two HDIT102 Fab molecules and one HDIT101 Fab molecule to the gB trimer (left) could be easily separated from homogenic HDIT102 binding to gB trimers by the perpendicular orientation of HDIT101 and HDIT102 Fabs when bound to gB

To determine the exact epitopes of HDIT101 and HDIT102 in gB, we solved the cryo-EM co-structures of Fab bound to HSV-1F or HSV-2G recombinant gB ectodomain in post-fusion conformation at resolutions of 3.12Å to 3.45Å (Fig. S3). The Fab fragments of HDIT101 and HDIT102 both bound to domain I of gB (HSV-1F/HSV-2G amino acids 154–364/146–356), indicating a shared epitope region (Fig. 3A, B, C and Fig. S4A, B, C). When we compared the cryoEM structure of HSV-1 gB solved in complex with HDIT101 Fab or HDIT102 Fab with the published x-ray crystal structure PDB: 2GUM [27] using the pairwise structure alignment tool TM-align [28], we observed a TM-score of 0.96 and 0.99, respectively, demonstrating almost identical structures (Fig. S4D, E, F). Our cryo-EM structure revealed the structural details of the HSV-1 gB amino acid residues T331-T337, which were not resolved in the X-ray crystal structure or by two other cryoEM structures (PDB: 7UI0 and PDB:7UHZ) [29]. The region L460-A490 which is bearing the most differences between HSV-1 and HSV-2 gB and was not resolved by x-ray crystallography could also not be resolved by cryoEM, suggesting high flexibility. We next compared the HSV-1F gB structure with HSV-2G gB when bound either by HDIT101 Fab or HDIT102 Fab (Fig. S4 G, H). The TM-score of HSV-1F gB vs. HSV-2G gB structures when HDIT101 Fab or HDIT102 Fab bound was 0.98 and 1.00, respectively, demonstrating that HSV-1F and HSV-2G gB ectodomains obtained almost identical protein structures in the post-fusion conformation, despite 24 amino acids are different between HSV-1 and HSV-2 gB sequences (Fig. S4I).

We next investigated which gB residues were in close proximity of the HDIT101 and HDIT102 complementary determining regions (CDRs). HDIT101 heavy and light chain CDR residues were in close proximity with HSV-1F/HSV-2G gB residues Y303/Y295, R304/R296, E305/E297, H308/H300 and D323/D315, while HDIT102 CDR residues were near HSV-1F/HSV-2G gB residues D199/D191, K204/K196, R304/R296, K320/K312, Y326/Y318 and R335/R327 (Fig. 3D, E and Fig. S4J, K).

HSV-2 gB Y295 made polar contact with a distance of 2.8Å with HDIT101 HC CDR2 N54 and D56 (Fig. S5A). HSV-2 gB E297 made polar contact with HDIT101 LC CDR1 Y32 and gB D315 with gB R296 and HDIT101 LC CDR1 H30a, thereby positioning R296 in proximity to HDIT101 LC CDR1 H30a, LC CDR1 Y32, LC CDR3 W96 and HC CDR3 Y97 (Fig. S5B). HSV-2 gB H300 formed non-polar bonds with the peptide-backbone of HC CDR1 S31a and CDR3 G98 (Fig. S5C). In the HDIT102 co-structure HSV-2 gB D191 made polar contact at 2.8Å and 2.7Å distance with HC CDR3 T100a and T100b, respectively (Fig. S5D). HSV-2 gB K196 made polar contact at 2.5Å distance with LC CDR2 D51 (Fig. S5E) and gB R327 formed polar contact with LC CDR2 D53 and non-polar contact with LC CDR2 Y50 (Fig. S5F). HSV-2 gB Y318 made polar contact with HC CDR3 D101 (Fig. S5G). The distance between HSV-2 gB R296 to HDIT102 CDR residues was > 3.5Å and no polar bonds were observed.

As the cryoEM structure showed that the epitopes of HDIT101 and HDIT102 have one shared amino acid at position (HSV1/HSV2 R304/R296) we investigated if the epitopes of HDIT101 and HDIT102 allow simultaneous binding of both IgGs and performed competition assays. Using the Fab fragments of HDIT101 and HDIT102 in an ELISA based assay, HDIT101, even when present at a 100-fold molar excess, did not displace HDIT102 from binding to either HSV-1 gB or HSV-2 gB, which is very likely due to the extremely slow dissociation rate of HDIT102. Using HEK293T cells ectopically expressing HSV-1F gB increasing concentrations of either HDIT101 IgG or HDIT102 IgG but not an irrelevant control IgG were able to reduce the binding signal of the murine MAb2c containing identical CDRs to HDIT101. These results indicate that steric hindrance prevents HDIT101 and HDIT102 from simultaneous binding to their epitopes (Fig. 3F). Interestingly, structural analyses showed that both Fabs bound in a perpendicular way, suggesting that inter-gB-trimer crosslinking by HDIT101 and HDIT102 IgG may result in differently oriented Fc domains and that heterogenic immune complexes may be formed by mixed binding of HDIT101 and HDIT102 to individual protomers within a trimer. To analyze this, a 1:1 molar mixture of HDIT101 and HDIT102 Fabs was incubated with recombinant HSV-1 gB trimers and 2D classes were calculated from cryo-EM micrographs. While the majority of classes showed homogenic immune complexes of three HDIT102 Fab molecules bound per gB trimer, heterogenic complexes with two HDIT102 and one HDIT101 Fab molecule could be easily identified based on the perpendicular orientation of HDIT101 and HDIT102 Fabs when bound to gB (Fig. 3G). We detected only heterogenic trimers consisting of two HDIT102 Fabs and one HDIT101 Fab. Neither trimers with two HDIT101 Fabs and one HDIT102 Fab nor homogenic trimers consisting of three HDIT101 Fab molecules were observed. We did not observe more than three Fab molecules bound per gB trimer which is in line with overlapping epitopes and the competition of binding to a single gB protomer. The data suggests that each protomer within the trimeric gB may be bound by either HDIT101 or HDIT102 and in the presence of both, heterogenic immune complexes may be formed.

### Signatures of evolutionary pressure by HDIT102-like endogenous IgG on HSV-2, but not HSV-1

To verify the structural data that was revealed using cryo-EM, HSV-1F or HSV-2G were propagated in Vero cells for several rounds with increasing concentrations of either HDIT101 or HDIT102. After five rounds of replication, presence of suboptimal concentrations of HDIT101 generated resistance mutant HSV-1F R304Q and HSV-2G R296Q, respectively, while propagation of the viruses under increasing doses of HDIT102 generated resistance escape mutants HSV-1F R335Q and HSV-2G R327W, respectively (Fig. S6A). We confirmed the importance of the identified gB residues in HDIT101 or HDIT102 induced inhibition of fusion using a flow cytometry-based fusion assay. gB amino acids conferring resistance and amino acids identified to be in close proximity to Fab residues in the cryo-EM structures were analyzed by alanine-substitution in the fusion assay, confirming the importance of HSV-1F gB D199, K204, Y326 and R335 as interface residues for HDIT102- and Y303, R304, E305, H308 and D323 as interface residues for HDIT101-induced fusion inhibition (Fig. S6B-E).

We next analyzed 451 HSV-1 and 368 HSV-2 full-length gB DNA sequences retrieved by nucleotide BLAST (NCBI) and found that HSV-1/2 R304/R296, Y303/Y295, E305/E297, H308/H300 and D323/D315 were conserved to 100%, suggesting that the HDIT101 targeted gB region is not under natural selection pressure neither in HSV-1 nor in HSV-2. In contrast, 451/451 (100%) of gB sequences from HSV-1 isolates had R335, however 55/368 (14.9%) of gB sequences from HSV-2 isolates possessed Q327, the same site that changed and developed resistance in vitro when HSV-2G was propagated in the presence of increasing doses of HDIT102 (Fig. S6F, G). Analysis for evolutionary positive selection using single-likelihood ancestor counting (SLAC) with *n* = 185 non-identical HSV-1 gB sequences and *n* = 201 non-identical HSV-2 gB sequences resulted for HSV-1 in 1 positively and 38 negatively selected sites and for HSV-2 gB in 6 positively and 13 negatively selected sites, suggesting that HSV-2 gB is under stronger selective pressure as compared to HSV-1 gB (Fig. S6H). When analyzing inferred non-synonymous and synonymous substitution rates for HSV-2 gB position 327 we found evidence for positive selection with dN-dS = 36.1 and p (dN/dS > 1) = 0.0375, suggesting that this site is indeed under positive selective pressure [25]. Mapping Q327 in a phylogenetic tree suggests a possible fixation of Q327 in branches (Fig. S6I), arguing that Q327 is evolving from R327. The data suggest that natural HDIT102-like antibodies exert immunologic pressure towards HSV-2, but not HSV-1, demonstrating suitability of HDIT102 as therapeutic candidate. Of note, we were unable to generate double resistant mutants of HSV-1F or HSV-2G when the viruses were propagated under suboptimal concentrations of a 1:1 molar mixture of HDIT101 and HDIT102 IgG in Vero cells. We also tried to generate double resistant mutants by starting with either HDIT101- or HDIT102-resistant viruses and propagation in suboptimal increasing concentrations of the respective other antibody, without success. Cloning of an HSV-1 gB mutant containing both, resistance change R304A (HDIT101) in combination with resistance change R335A (HDIT102), resulted in a functionally active gB protein showing fusion activity in the cell-to-cell fusion assay, which was resistant to both HDIT101 as well as HDIT102 treatment (data not shown). Together these data suggest that a double resistant mutant gB could be functionally active, however may not evolve readily in the context of virus replication.

### The Fc effector function of HDIT101 and HDIT102 to mediate HSV-1 phagocytosis by monocyte-derived macrophages and dendritic cells likely contributes to in vivo efficacy

**Fig. 4:**
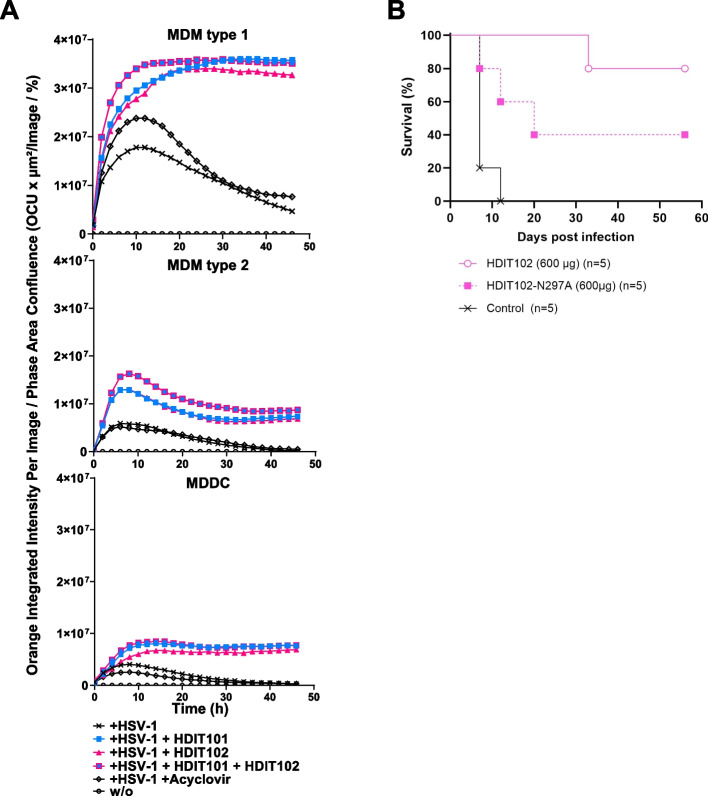
HDIT102 induces phagocytosis by myeloid cell types and the Fc effector function likely contributes to in vivo efficacy. **A** CD14 + monocytes were isolated from PBMCs and differentiated for one week to monocyte-derived macrophages (MDM) or monocyte-derived dendritic cells (MDDC) by adding GM-CSF (MDM type 1), M-CSF (MDM type 2), or GM-CSF + IL-4 (MDDC). The differentiated cells were then exposed to HSV-1 labelled with a pH-sensitive dye with or without the addition of HDIT101 and/or HDIT102 antibody or acyclovir. Three technical replicates each were then monitored using an Incucyte system (Sartorius) at intervals of 1h. The amount of virus taken up as measured by dye fluorescence was normalized to the cell count in the imaged area. Representative results for one of three independent PBMC donors are shown. **B** Eight-week-old immunocompetent female BALB/cOlaHsd mice (*n* = 5, each group) were infected intravaginally with HSV-2G using a lethal dose of 5 × 10^4^ TCID50. Subsequently, 600 μg of HDIT102 or HDIT102-N297A mutant were injected intraperitoneally, while the control group received no treatment. The statistical differences between survival curves were calculated using Logrank Mantel Cox test. *p* = 0.15 (HDIT102 vs. HDIT102-N297A). *p* = 0.0016 (HDIT102 vs. untreated). *p* = 0.03 (HDIT102-N297A vs. untreated)

The efficacy of antibodies in therapeutic application often not solely depends on their binding capacity but also on the Fc-mediated effector function. To better understand the effects of how HDIT101 and HDIT102 work, we first tested whether both antibodies would induce antibody-dependent cellular cytotoxicity (ADCC) or complement-dependent cytotoxicity (CDC). We found that neither HDIT101, nor HDIT102, induced ADCC or CDC (Fig. S7 and data not shown). We next tested whether the antibodies would mediate phagocytosis of virus particles (ADCP) by antigen presenting cells (APCs). Human monocyte-derived macrophages (MDM) of type 1, type 2, or dendritic cells (MDDC) were generated from PBMCs and present a suitable primary model to test Fc-effector functions in cell culture. To trace the virus, we labelled the virus with a pH-sensitive dye. The cells were incubated with labelled virus, either without antibodies or in the presence of either HDIT101, HDIT102, or a 1:1 molar mix of both antibodies. The fluorescent signal indicates uptake into acidic endosomes and the impact of the investigated antibody on cellular uptake could be investigated. HDIT101 as well as HDIT102 exerted an enhanced uptake of virus particles into acidic endosomes within ten hours as compared to virus alone or virus in combination with acyclovir. The combination of both antibodies showed no substantial enhancement over using the individual antibodies (Fig. 4A). The data suggest that the HDIT101 and HDIT102 Fc domain contributes to promoting ADCP of viral particles. Fc-dependent uptake of viral immune complexes may hence impact also the in vivo efficacy of the investigated antibodies.

To elaborate on this further, we generated a commonly used IgG Fc domain mutant HDIT102 version containing the N297A substitution in the Fc domain, a change which substantially decreases Fc-gamma dependent activity and analyzed the requirement for a functional Fc domain for efficient therapeutic treatment in vivo. Immunocompetent female BALB/cOlaHsd mice were infected intravaginally with HSV-2G and treated with the same dose of wild type HDIT102, or HDIT102-N297A mutant antibodies, and disease development and survival was monitored. The results show a substantially longer survival of HDIT102 wild type as compared to HDIT102-N297A treated mice, suggesting that the Fc domain and interaction with Fc-receptors may be implicated in the in vivo efficacy (Fig. 4B). Similar data was obtained for HDIT101 (data not shown). Of note, HDIT102-N297A treated mice were still protected to some degree as compared to control mice, potentially explained by some remaining ADCP activity of the N297A mutant, as has been described in earlier studies [30–32].

### HDIT101 as well as HDIT102 increase T-cell activation after ADCP by macrophages

**Fig. 5:**
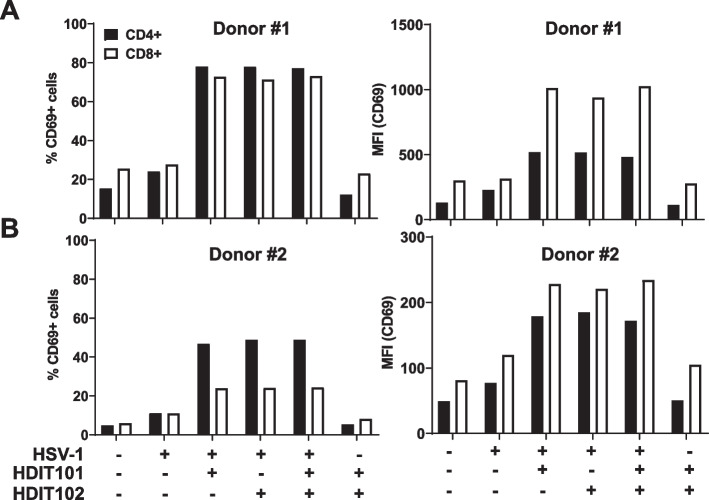
HDIT102-induced phagocytosis of HSV-1 by MDMs activates autologous T-cells. Autologous T-cell activation in the CD14- fraction of PBMCs was measured after exposure to CD14 + monocyte derived macrophages (type 1) from two independent HSV-seropositive healthy donors, (**A**) donor 1, (**B**) donor 2, that were pre-stimulated for 24 h with HSV-1F (MOI 10) in the presence or absence of neutralizing amounts of HDIT102 or HDIT101 or a 1:1 combination of both. Analysis of activation was done by measuring CD69 expression on CD4 + and CD8 + cell populations using flow cytometry. Percentage of CD69 + cells as well as mean fluorescent intensity were measured and analyzed separately

Data from the clinical program for HDIT101 suggested that modulation of the immune responses could be involved in the HDIT101-mediated effects. Since HDIT102, as well as HDIT101, were able to enhance uptake of viral particles into acidic endosomes of APCs in vitro and the Fc domain contributed to efficacy in mice, we speculated that this could enhance also T-cell responses. T-cells are binding to APC-processed peptides presented via MHC-I and MHC-II. We tested the hypothesis that enhanced phagocytosis of HSV particles by HDIT101 and/or HDIT102 may lead to an enhanced antiviral T-cell response. To do that MDM type 1 was generated from HSV-1 seropositive donors and incubated with either HSV-1F alone or in combination with HDIT101, HDIT102 or both antibodies together. As negative control, the cells were incubated with both antibodies in the absence of virus or left untreated. Twenty-four hours after stimulation, autologous T-cells (CD14- fraction) were added and after further 24 h the CD4 + and CD8 + cell populations were analysed for activation by measuring CD69. In two independent donors the stimulation of MDMs with immune complexes of HSV-1F with either HDIT101, HDIT102 or HDIT101 + HDIT102 induced CD69 expression on the surface of both, CD4 + and CD8 + cells (Fig. 5A, B). While the percentage of CD69 + cells was higher in the CD4 + cell population, the MFI of CD69 was higher in the CD69 + CD8 + cell population, suggesting that a higher fraction of CD4 + than CD8 + T-cells becomes activated, but that activation strength as measured by CD69 quantity per cell is higher in the responsive CD8 + T-cells. Together the data demonstrate that both antibodies alone or in combination are capable of mediating phagocytosis that may result in an enhanced activation of T-cells in vitro.

### The combination of HDIT101 and HDIT102 exerts synergistic therapeutic effects in vivo

**Fig. 6:**
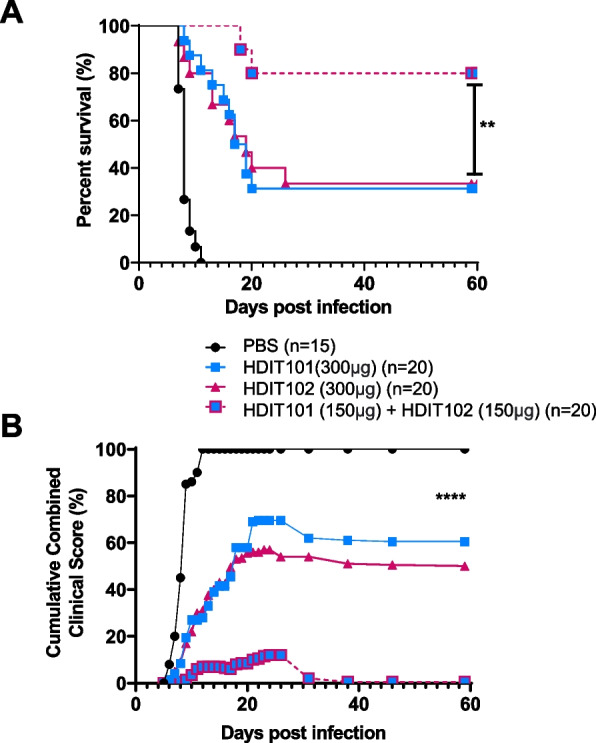
Combination therapy with HDIT101 and HDIT102 exerts synergistic effects in vivo. Eight-week-old BALB/c mice were infected with a lethal dose of HSV-2G (5 × 10.^4^ TCID50). HDIT102 was mixed with HDIT101 at equimolar ratio (combination therapy) and injected at a final total IgG dose of 300 μg intraperitoneally. HDIT101 or HDIT102 alone (monotherapy) were injected intraperitoneally at the same dose (300 μg). The graphs show combined results from three independent experiments. In total, 20 mice per treatment group and 15 mice for the control arm were used. Survival (**A**) and clinical symptoms (**B**) were scored for a period of 60 days. The statistical differences between survival curves were calculated using Logrank Mantel Cox test. **p = 0.0069. The differences between cumulative combined clinical scores were analyzed using Kolmogorov-Smirnow test. ****p < 0.0001

We did not observe general synergistic effects when comparing the HDIT101 and HDIT102 combination treatment with the individual antibodies at equal IgG quantities in any of the in vitro assays tested, including neutralization, cell-to-cell spread, ADCP and T-cell activation by stimulated APCs. To analyze the combination and possible synergy of both IgGs in vivo immunocompetent BALB/cOlaHsd mice were infected intravaginally with HSV-2G and treated four hours later with either 300 μg HDIT101, or 300 μg HDIT102 or with a combination of 150 μg HDIT101 + 150 μg HDIT102 and survival as well as clinical scores were monitored over time. Surprisingly, while for both HDIT101 or HDIT102 treatment groups half of the mice did not survive by day 60, the combination of both IgGs resulted in a significantly longer survival at the same total IgG dose, rescuing 90% of the animals, indicating that both antibodies induce a synergistic therapeutic effect in vivo (Fig. 6A). When comparing the cumulative combined clinical scores for the treated animals the benefits of the combination of HDIT101 and HDIT102 on symptom development are already visible in the early days after treatment, demonstrating also synergistic effects on prevention of symptom development (Fig. 6B). Together the data show, that the combination of HDIT101 and HDIT102 IgG exerted synergistic effects in the acute intravaginal HSV-2 infection of immunocompetent BALB/cOlaHsd mice, suggesting that the combination may be an attractive new drug product.

## Discussion

Monoclonal antibodies show potential as emerging therapeutics for managing viral infections. Based on the high medical need for novel viable therapeutics in HSV associated disease conditions, we have developed the fully human antibody HDIT102. Similar to the humanized antibody HDIT101, which we have previously employed in clinical phase I and II trials, HDIT102 targets domain I of the fusion protein gB of HSV-1 and HSV-2. Domain I is part of the outermost exposure on the gB surface in the prefusion conformation and contributes to the structural rearrangements that gB undergoes during the fusion process, making it an attractive target for antibody therapy. HDIT102 demonstrated superior binding characteristics to HSV-2 gB as compared to the humanized antibody HDIT101, while showing similar efficiency in neutralizing cell-free virus and blocking cell-to-cell spread in vitro. Cell-to-cell spread inhibition is likely the main way of viral spread in vivo and it was proposed for HSV-1 that individuals with higher levels of cell-to-cell spread inhibiting antibodies may have fewer orolabial recurrences [26], suggesting that this characteristic may be advantageous when developing a monoclonal antibody therapy against HSV. Cell-to-cell spread of HSV has been considered as viral immune evasion mechanism [33]. In fact, also for other viruses, e.g. SARS-CoV-2 it was shown that cell-to-cell transmission is more refractory to neutralizing antibodies and convalescent plasma [34]. HSV gB and other HSV glycoproteins have been proposed to be transported to the cell membrane and re-imported via endosomes in a Rab-dependent way before becoming incorporated into the viral membrane within the trans-Golgi network [35]. The data provided here suggest that both HDIT101 and HDIT102 bind to gB exposed at the cell-surface of infected cells and are co-internalized and transported on the natural gB trafficking way, hence interact with gB before becoming incorporated into the viral membrane, blocking spreading of newly produced progeny viruses.

For structural analysis of gB as druggable target and to identify the epitopes of both antibodies we utilized cryoEM. We have solved here for the first time the cryoEM structure of the HSV-2 gB ectodomain in complex with two different Fabs binding to domain I at a resolution between 3.1–3.5Å. We also solved the HSV-1 gB ectodomain cryoEM structure in complex with the two different Fabs which revealed very high structural similarity to the published x-ray crystal structure (PDB: 2GUM, [27]) or cryoEM structures (PDB: 7UI0 and PDB:7UHZ) [29]. We observed very high similarity in the solved HSV-2 gB ectodomain structure to HSV-1 gB, despite 24 amino acid differences, however, this may not be surprising given the overall strong conservation of gB postfusion structural organization within the Herpesviridae [36–39]. There are major structural rearrangements between the pre- and postfusion conformation of HSV1 gB [40, 41]. However, the amino acid residues near the HDIT101 and HDIT102 CDRs in the post-fusion conformation are all solvent exposed in the pseudoatomic model of the prefusion conformation of glycoprotein B of Herpes simplex virus 1 (PDB-ID: 6Z9M), suggesting that their accessibility does not preclude the potential binding of one of the two Fabs, HDIT101 and HDIT102.

As both antibodies target conserved regions of gB, we investigated occurrence of mutations within the antibody epitopes. While for each antibody treatment alone, resistant viruses with single amino acid substitutions HSV-1 gB R304Q (HDIT101), HSV-2 gB R296Q (HDIT101), HSV-1 gB R335Q (HDIT102) and HSV-2 gB R327W (HDIT102) grew out in a small number of rounds of replication under suboptimal antibody concentrations in vitro, this was not the case when using an antibody mix containing a 1:1 molar ratio of both IgGs, suggesting that targeting overlapping but not identical epitopes with two different monoclonal antibodies may confer a stronger pressure on the virus, possibly due to structural constraints in the targeted gB region. Quite surprisingly, when analyzing public gB sequences of clinical isolates, we found signs for evolutionary pressure of HDIT102-like endogenous antibodies on HSV-2 isolates, but not on HSV-1. To our knowledge this is the first sign for differences in the immunologic response against gB and evolutionary immune escape between HSV-1 and HSV-2. Nearly 15% of HSV-2 gB sequences had glutamine at gB amino acid position 327, a position that conferred HDIT102-resistance in vitro when changed from R327 in an alanine scan, while 100% of HSV-1 isolate gB sequences had arginine at this position (R335) suggesting that naturally HDIT102-like antibodies exert immunological pressure on HSV-2, but surprisingly not on HSV-1. Given that HDIT102 was derived from an scFv library in which VH and VL are artificially shuffled, it is possible that natural HDIT102-like antibodies target the same residue in gB, but may bind slightly differently, leading to the natural change R327Q as compared to HDIT102-induced in vitro substitution R327W.

Therapeutic effects by HDIT101 in vivo have been shown before [17]. In this previous work the efficacy of HDIT101 was tested only in an immunodeficient HSV-1 NOD/SCID mouse model and administered at 3 single dose levels between 2,5mg/kg and 15mg/kg, resulting in an 80% cure rate at the highest dose level. The murine ancestor of HDIT101, mAb 2c, has, however, been shown to neutralize HSV-2 less efficiently than HSV-1 [42]. We have shown here that a combination therapy with HDIT101 and HDIT102 exerts potent synergistic antiviral effects in an acute HSV-2 intravaginal infection mouse model. Combinations with therapeutic antibodies showing no signs of synergism in vitro but exerting potent therapeutic synergy in vivo have been investigated for other viral diseases before, e.g. Chikungunya virus [43]. Of note however, we have shown here that the combination of two antibodies binding to neighbouring epitopes in the same structurally confined region of the trimer-forming glycoprotein B of herpes simplex virus, can lead to synergistic effects in vivo. In Cryo-EM analyses we did not detect more than three Fab molecules binding to a single gB ectodomain trimer when incubation with a 1:1 HDIT101 + HDIT102 Fab mix, strongly arguing for steric hindrance for binding of HDIT101 to its epitope if HDIT102 has occupied its epitope on the same gB protomer and vice versa. The majority of trimeric gB ectodomain in post-fusion conformation was bound homogenically by three HDIT102 Fab molecules, as shown in cryo-EM 2D images. However also heterogenic structures with two HDIT102 and one HDIT101 Fab molecules could be identified. When considering different binding kinetics of the antibodies (for HDIT102 an extraordinarily low off rate after gB binding) it is conceivable that heterogenic interaction of two different antibodies with trimeric HSV gB could lead to irregularly cross-linked immune complexes that after processing may be more immunogenic in vivo and may well explain the synergistic effects observed in the combination of HDIT101 and HDIT102 in vivo. In this regard, the perpendicular orientation of both Fabs when bound to trimeric gB may play a role. Our findings could potentially be relevant for other multivalent proteins, such as trimeric fusion proteins with multiple epitopes available, the concept behind this idea, however, relies on multimeric protein targets that can form heterogenic immune complexes when bound by different antibodies at each protomer. Despite our attempts we were unable to recapitulate the synergistic effects seen in vivo by any tested in vitro assay. It is hence conceivable that the presence of important immunological components, which are lacking in the in vitro assay, may explain the synergistic effects observed in vivo.

Most interestingly, despite both HDIT101 and HDIT102 are potently inducing antibody-dependent phagocytosis (ADCP), they are not capable of mediating ADCC or CDC, it is imperative to acknowledge that this activity was observed in vitro using human effector cells. Nonetheless, our interpretation remains valid, as demonstrated by the mouse in vivo model. While it is crucial to recognize interspecies differences, it is also noteworthy to acknowledge the substantial functional homology of ADCP between humans and mice, supported by similarities in Fc receptors and Fc affinities. Of note, anti-HSV antibodies mediating antibody-dependent cellular cytotoxicity (ADCC) have been connected to superior protection [44–46]. Low levels of ADCC-mediating antibodies were suggested to play an important role for the absent protection by vaccines against HSV-2 [47, 48]. The role of antibody-mediated cellular phagocytosis (ADCP) and subsequent immune cell activation in the immune response to HSV-1/2 infection, in particular T-cell activation, has not been investigated in depth [47, 49]. ADCP is a known Fc-effector function and has been proposed to be implicated in anti-HSV effects observed in vaccine trials [47]. Indeed, ADCP has also been recently proposed as possible main function of anti-cancer monoclonal antibody therapies, suggesting a more widespread and understudied part of monoclonal antibody effector functions [50]. As consequence of induced ADCP by HDIT101 and HDIT102, antigen-presenting cells may conceivably process antibody-HSV complexed particles and subsequently recruit CD4 + and CD8 + T-cells to MHC presented viral peptides. Changes from the conventional proteasome to the immunoproteasome and alterations in the MHC-I and MHC-II immunopeptidome during proinflammatory conditions have been described [51, 52]. Likewise, MHC-independent activation of T-cells may also play a role. While we did not observe quantitative differences in autologous T-cells activated in the presence of HDIT101, HDIT102 or a combination of both in vitro, a combination of different monoclonal antibodies may induce quantitative or qualitative differences in vivo, which may account for the synergistic antiviral effects of a combination therapy with HDIT101 and HDIT102 observed in vivo. Future studies should determine the APC-presented MHC-immunopeptidome after ADCP in absence or presence of different monoclonal antibodies and combinations or differences in TCR-clonalities of activated T-cell subsets.

In conclusion, we have shown that the fully human antibody HDIT102 has great potential for further clinical development as a potent novel HSV therapeutic particularly in combination with its clinical humanized ancestor antibody HDIT101.

## Conclusion

The combination of two monoclonal antibodies for the treatment of chronic HSV-2 may provide a novel therapeutic option. Antibody characteristics to inhibit cell-to-cell spread, to mediate uptake of cell free-viruses by phagocytic cells and concomitantly stimulate T-cell responses may promote cellular immunity and may have benefits in preventing recurrences.

### Supplementary Information

Supplementary Material 1. Fig. S1. HDIT102 IgG does not cross-react with other herpesviruses than HSV-1/2 and has a very low dissociation rate (kdis). (A) Binding of a dilution series of HDIT102 at several concentrations to HSV-1/2, VZV, HCMV and EBV antigens was analyzed by ELISA using microplates coated with respective viral antigens (Enzygnost, Siemens). Absorbance at 450 nm was measured. Anti-HSV-ELISA does not discriminate between detection of anti-HSV-1 and anti-HSV-2 IgGs. Binding was detected with an HRP-conjugated anti-human gamma Fc-specific IgG. Cytotect (anti-CMV polyclonal antibody preparation) was used as a positive control for all. (B) HDIT101 IgG was tested in biolayer-interferometry against immobilized HSV-1F gB. (C) HDIT102 IgG was tested in biolayer-interferometry against immobilized HSV-1F gB. Fig. S2. HDIT102 efficiently inhibits cell-to-cell spread of HSV-1F in Vero cells. Inhibition of HSV-1F cell-to-cell spread by HDIT102. Fluorescence microscopy images of Vero cells infected with HSV-1F and subsequently treated with either HDIT102, HDIT101, human polyclonal anti-HSV antibody or left untreated. Plaque formation was visualised by anti-HSV immuno- and Hoechst staining. Representative images are shown. Arrows show plaques or initially infected cells. Fig. S3. Cryo-EM data analysis of co-structures of trimeric HSV-1F and HSV-2G gB. (A) - (D) The plots show the Fourier shell correlation (FSC) curves of the final calculated density map (black) and the FSC curve calculated between the final map and the atomic model (grey). The reported resolutions for the maps is based on the “gold-standard” FSC = 0.143 criterion and FSC = 0.5 for the FSC between map and model. (E) - (H) The final 3D reconstructions are shown in three different views (top, side and bottom) and colored according to the local resolution calculated using the ResMap [53] implementation in RELION 4.0 [20]. Fig. S4. HSV-1 gB cryo-EM co-structure with HDIT101 or HDIT102 Fab. (A) Side, bottom and top view of co-structure of trimeric HSV-1F gB in post-fusion conformation (green) with three HDIT101 Fab molecules (blue) solved by cryo-EM at a resolution of 3.27 Å are shown. (B) The co-structure of HDIT102 Fab (magenta) bound to trimeric HSV-1F gB in post-fusion conformation (green) was determined at a resolution of 3.44 Å and side, bottom and top views are shown. (C) Overlay of the co-structures indicating overlapping epitopes of HDIT101 and HDIT102 Fabs on HSV-1F gB indicating perpendicular orientation of both Fabs to another. (D) Overlay of published x-ray crystal structure (PDB:2GUM) with HSV-1 cryoEM gB structure derived in complex with HDIT101 Fab. (E) Overlay of published x-ray crystal structure (PDB:2GUM) with HSV-1 cryoEM gB structure derived in complex with HDIT102 Fab. (F) Overlay of HSV-1 cryoEM gB structure derived in complex with HDIT101 Fab and HDIT102 Fab. (G) Overlay of HSV-1 and HSV-2 cryoEM gB structures derived in complex with HDIT101 Fab. (H) Overlay of HSV-1 and HSV-2 cryoEM gB structures derived in complex with HDIT102 Fab. (I) Overlay of HSV-1 and HSV-2 cryoEM gB structures derived in complex with HDIT102 Fab with differing amino acid residues marked in magenta. (J) Detailed image of the HSV-1 gB residues interacting with HDIT101 (compare with Fig. 3D). (K) Detailed image of the HSV-1 gB residues interacting with HDIT102 (compare with Fig. 3E). Fig. S5. HSV-2 gB interactions with HDIT101 or HDIT102 CDR amino acid residues. HSV-2 gB residues within 4Å distance of residues in the HDIT101 or HDIT102 CDRs were determined by Pymol and subsequently analyzed individually for polar (red) or non-polar (yellow) interactions. Oxygen groups are highlighted in red, nitrogen groups in blue. Carbon atoms involved in non-polar interactions were not coloured. (A)-(C) Interaction of HDIT101 CDR residues with HSV-2 gB residues in the binding interface. (D)-(G) Interaction of HDIT102 CDR residues with HSV-2 gB residues in the binding interface. Fig. S6. Analysis of epitope residues conferring resistance to HDIT101 or HDIT102 induced fusion inhibition. (A) Amino acid alignment of HSV-1F and HSV-2G gB with indicated key residues conferring resistance to HDIT101 (HSV-1 gB R304Q/ HSV-2 gB R296Q) or HDIT102 (HSV-1 gB R335Q/ HSV-2 gB R327Q) evolving *in vitro*. (B) Cell-cell fusion assay and inhibition of selected HSV-1 gB mutants by HDIT102 IgG. Changes in fusion inhibition by HDIT102 were calculated comparing HDIT102 treated with untreated cells for each mutant and normalized to wild type gB (WT gB) mean values are shown for at least three independent biological replicates (*n*=3). Statistical analyses were performed using unpaired t-tests.  (C) Relative fusion activity of selected gB mutants compared to wild type protein. Fusion activity as a combined indicator of cell surface expression and fusogenicity of gB mutants was analyzed by comparison to wild type gB (WT gB) in the absence of any antibody. Mean values of three independent biological replicates are shown with error bars indicating standard deviation (*n*=3). Statistical analyses were performed using unpaired t-tests. (D) Cell-cell fusion assay and inhibition of selected HSV-1 gB mutants by HDIT101. Changes in fusion inhibition by HDIT102 were calculated comparing HDIT101 treated with untreated cells for each mutant and normalized to wild type gB (WT gB) mean values are shown for at least three independent biological replicates (*n*=3). Statistical analyses were performed using unpaired t-tests. (E) Relative fusion activity of selected gB mutants compare to wild type protein. Fusion activity as combined indicator of cell surface expression and fusogenicity of gB mutants was analyzed by comparison to wild type gB (WT gB) in the absence of any antibody. Mean values of three independent biological replicates are shown with error bars indicating standard deviation (*n*=3). Statistical analyses were performed using unpaired t-tests. 451 HSV-1 (F) and 368 HSV-2 (G) gB DNA sequences were retrieved from the NCBI sequence database by using nucleotide BLAST against HSV-1F or HSV-2G gB DNA sequences, aligned and analyzed for variations in the HDIT101 or HDIT102 epitope regions to study signatures of evolutionary pressure from natural HDIT102-like antibodies. The graphs illustrate the conservation at each epitope amino acid position in the gB proteins. The residue number is indicated in the x-axis and the percentage of conservation is indicated in the y-axis. (H) Single-likelihood ancestor counting analysis was performed on alignments of 185 full-length HSV-1 gB and 201 full-length HSV-2 gB sequences to identify positively selected residues. The difference in non-synonymous and synonymous substitution rates are shown on the y-axis and gB codon position in the alignment on the x-axis. (I) Phylogentic tree demonstrating HSV-2 gB isolates containing R327 or Q327. Fig. S7. HDIT102 does not elicit ADCC on HSV-1F or HSV-2G infected Vero cells or HEK293T cells ectopically expressing gB. Different dilutions of HDIT102 IgG or human polyclonal serum containing anti-HSV IgGs were added to HSV-1F or HSV-2G infected target cells, followed by incubation with effector Jurkat cells (Promega #G7010) stably expressing FcγRIIIA receptor, V158 (high affinity) variant and the NFAT-luciferase reporter at an effector-to-target cell ratio of 6:1. Infected Vero cells or HEK293T cells expressing either HSV-1 gB or HSV-2 gB were used as target cells. ADCC was quantified by luminescence readout from luciferase activity upon NFAT pathway activation. (A) ADCC activity of HDIT102 IgG or isotype control on Vero cells infected with either HSV-1F or HSV-2G or uninfected. Mean of three technical replicates is shown. (B) Same as in A, however using human polyclonal antibody as control. (C) Summary of A and B showing fold ADCC induction at highest antibody concentration. (D) ADCC activity of HDIT102 IgG on parental HEK293T or HEK293T stably expressing either HSV-1F gB or HSV-2G gB. Mean of three technical replicates is shown. (E) Same as in D, however using human polyclonal antibody as control. (F) Summary of D and E showing fold ADCC induction at highest antibody concentration. (G) and (H) Analysis of HDIT102-mediated complement dependent cytotoxicity by quantitative evaluation of the terminal C5b-9 complement complex. An ELISA was employed to measure supernatant levels of C5b-9 complex to quantify complement activation by HDIT102. HSV-1F or HSV-2G infected or uninfected Vero cells were incubated with heat-inactivated or with not heat-inactivated IgG-depleted human serum in the presence or absence of test antibodies (either HDIT102, human polyclonal IgG, or Cytotect as positive controls, or unrelated isotype IgG as negative control). Statistical analysis was done using two-way ANOVA, error bars represent standard deviation of the mean for two technical replicates, ****p< 0.0001)

## Data Availability

The structures and EM-maps have been deposited in the Protein Data Bank and Electron Microscopy Data Bank, respectively (PDB ID codes: 8RGZ, 8RH0, 8RH1, 8RH2; EMBD ID codes: 19163, 19164, 19165, 19166).
