# Supplementary material for: Development of a highly effective combination monoclonal antibody therapy against Herpes simplex virus
Source: J Biomed Sci. 2024 May 28;31:56. doi: 10.1186/s12929-024-01045-2 (PMC11134845; doi:10.1186/s12929-024-01045-2)
Supplement: Supplementary file 1 — Supplementary Material 1. Fig. S1. HDIT102 IgG does not cross-react with other herpesviruses than HSV-1/2 and has a very low dissociation rate (kdis). (A) Binding of a dilution series of HDIT102 at several concentrations to HSV-1/2, VZV, HCMV and EBV antigens was analyzed by ELISA using microplates coated with respective viral antigens (Enzygnost, Siemens). Absorbance at 450 nm was measured. Anti-HSV-ELISA does not discriminate between detection of anti-HSV-1 and anti-HSV-2 IgGs. Binding was detected with an HRP-conjugated anti-human gamma Fc-specific IgG. Cytotect (anti-CMV polyclonal antibody preparation) was used as a positive control for all. (B) HDIT101 IgG was tested in biolayer-interferometry against immobilized HSV-1F gB. (C) HDIT102 IgG was tested in biolayer-interferometry against immobilized HSV-1F gB. Fig. S2. HDIT102 efficiently inhibits cell-to-cell spread of HSV-1F in Vero cells. Inhibition of HSV-1F cell-to-cell spread by HDIT102. Fluorescence microscopy images of Vero cells infected with HSV-1F and subsequently treated with either HDIT102, HDIT101, human polyclonal anti-HSV antibody or left untreated. Plaque formation was visualised by anti-HSV immuno- and Hoechst staining. Representative images are shown. Arrows show plaques or initially infected cells. Fig. S3. Cryo-EM data analysis of co-structures of trimeric HSV-1F and HSV-2G gB. (A) - (D) The plots show the Fourier shell correlation (FSC) curves of the final calculated density map (black) and the FSC curve calculated between the final map and the atomic model (grey). The reported resolutions for the maps is based on the “gold-standard” FSC = 0.143 criterion and FSC = 0.5 for the FSC between map and model. (E) - (H) The final 3D reconstructions are shown in three different views (top, side and bottom) and colored according to the local resolution calculated using the ResMap [53] implementation in RELION 4.0 [20]. Fig. S4. HSV-1 gB cryo-EM co-structure with HDIT101 or HDIT102 Fab. (A) Side, bott [file 12929_2024_1045_MOESM1_ESM.pdf]

Figure S1

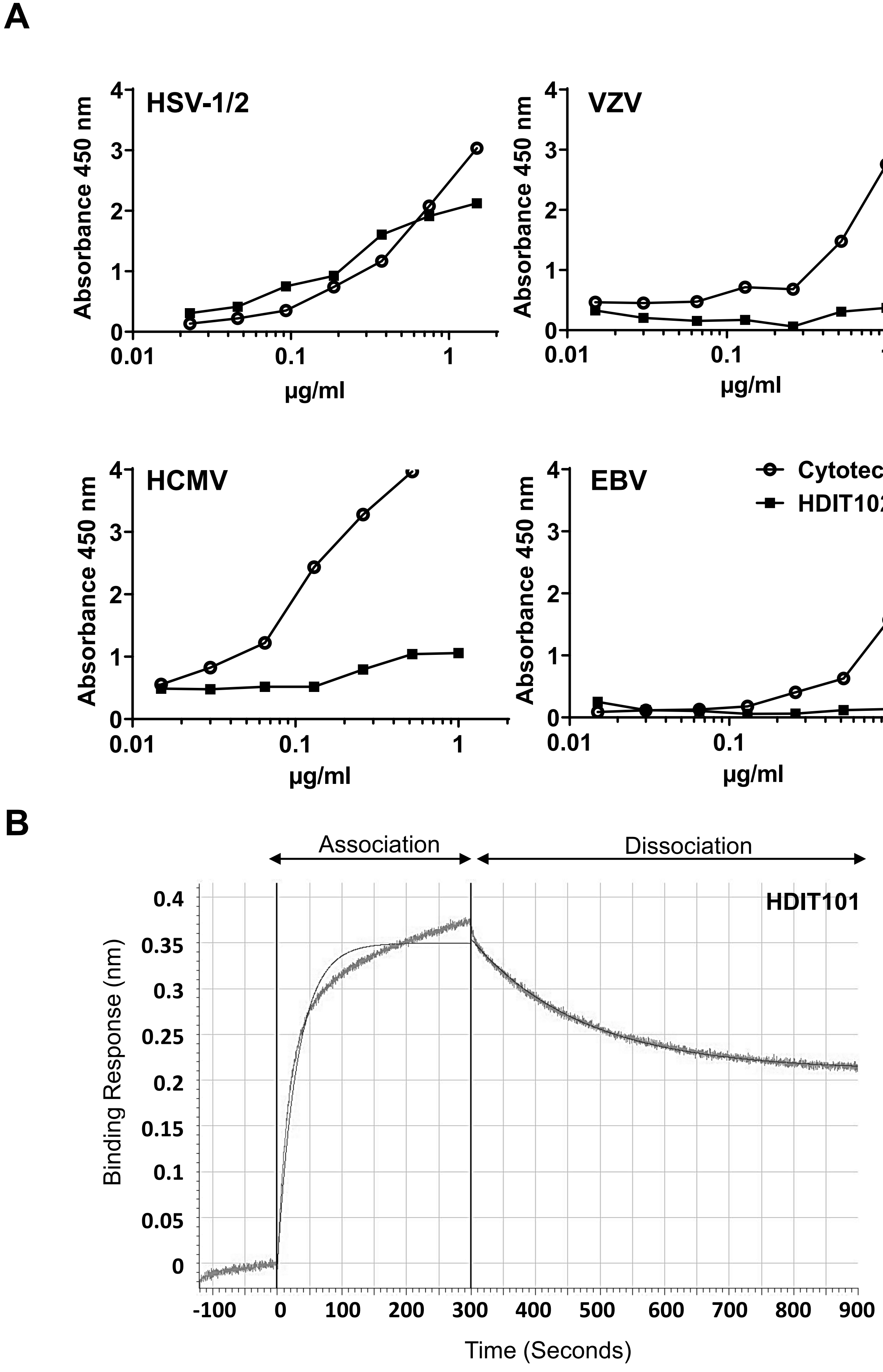

A
HSV-1/2
VZV
HCMV
EBV
Absorbance 450 nm
µg/ml
0.01
0.1
1
Cytotect
HDIT102
B
Association
Dissociation
HDIT101
Binding Response (nm)
Time (Seconds)

C

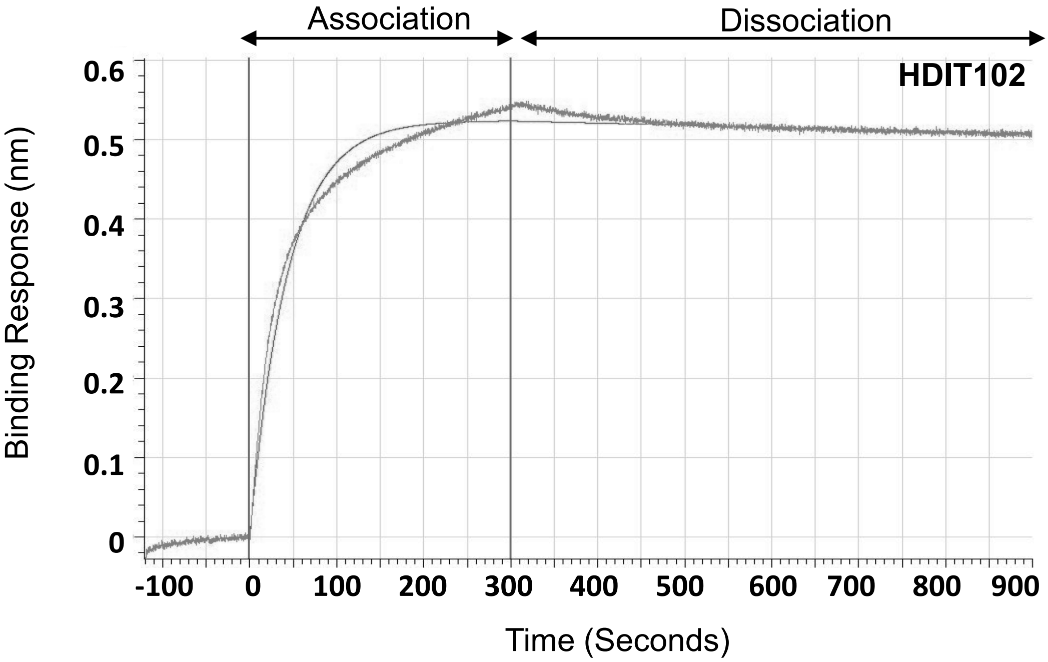
Association
Dissociation
HDIT102
Binding Response (nm)
0.6
0.5
0.4
0.3
0.2
0.1
0
-100
0
100
200
300
400
500
600
700
800
900
Time (Seconds)

Figure S2

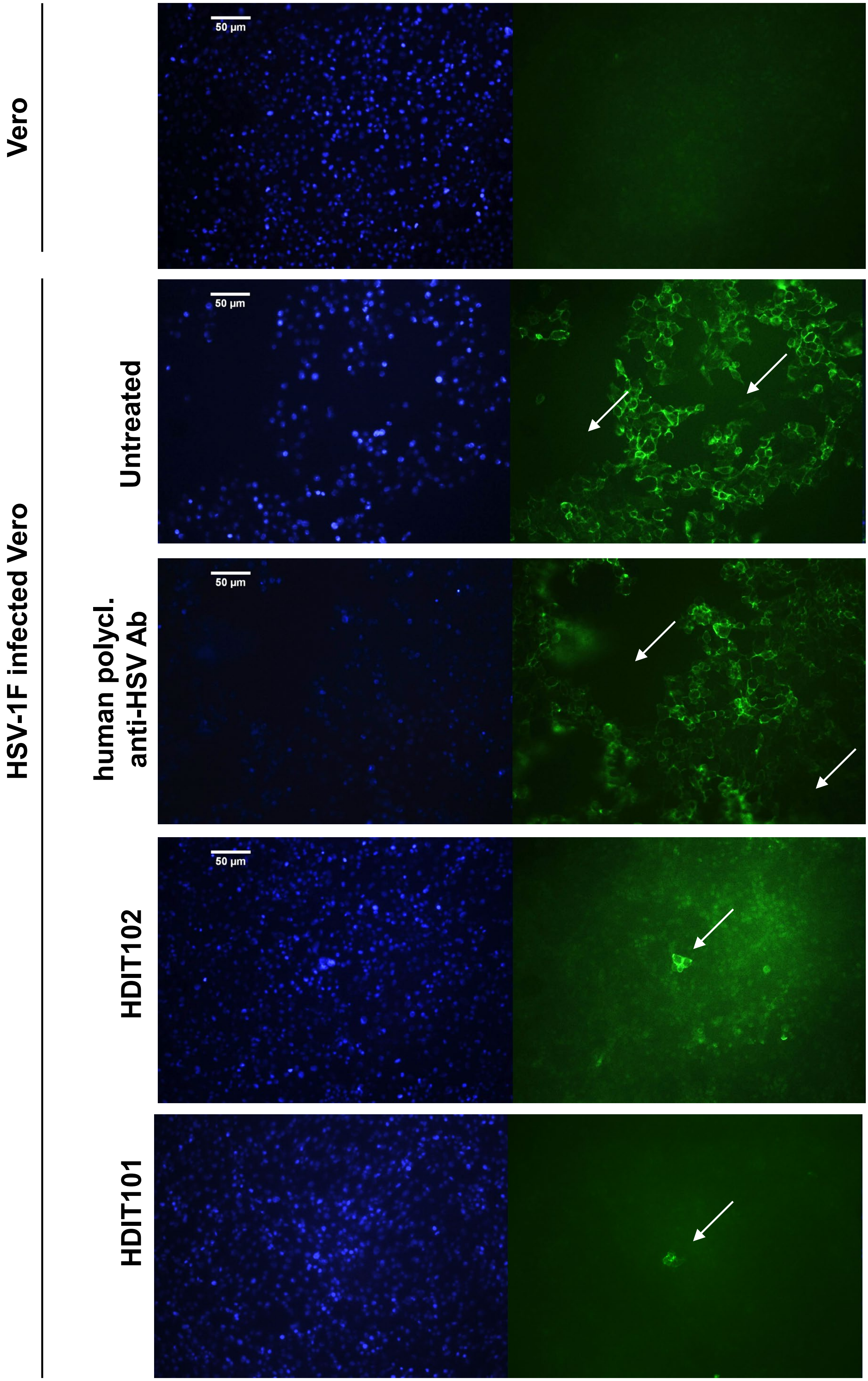

Vero
HSV-1F infected Vero
Untreated
human polycl. anti-HSV Ab
HDIT102
HDIT101
50 µm

Figure S3

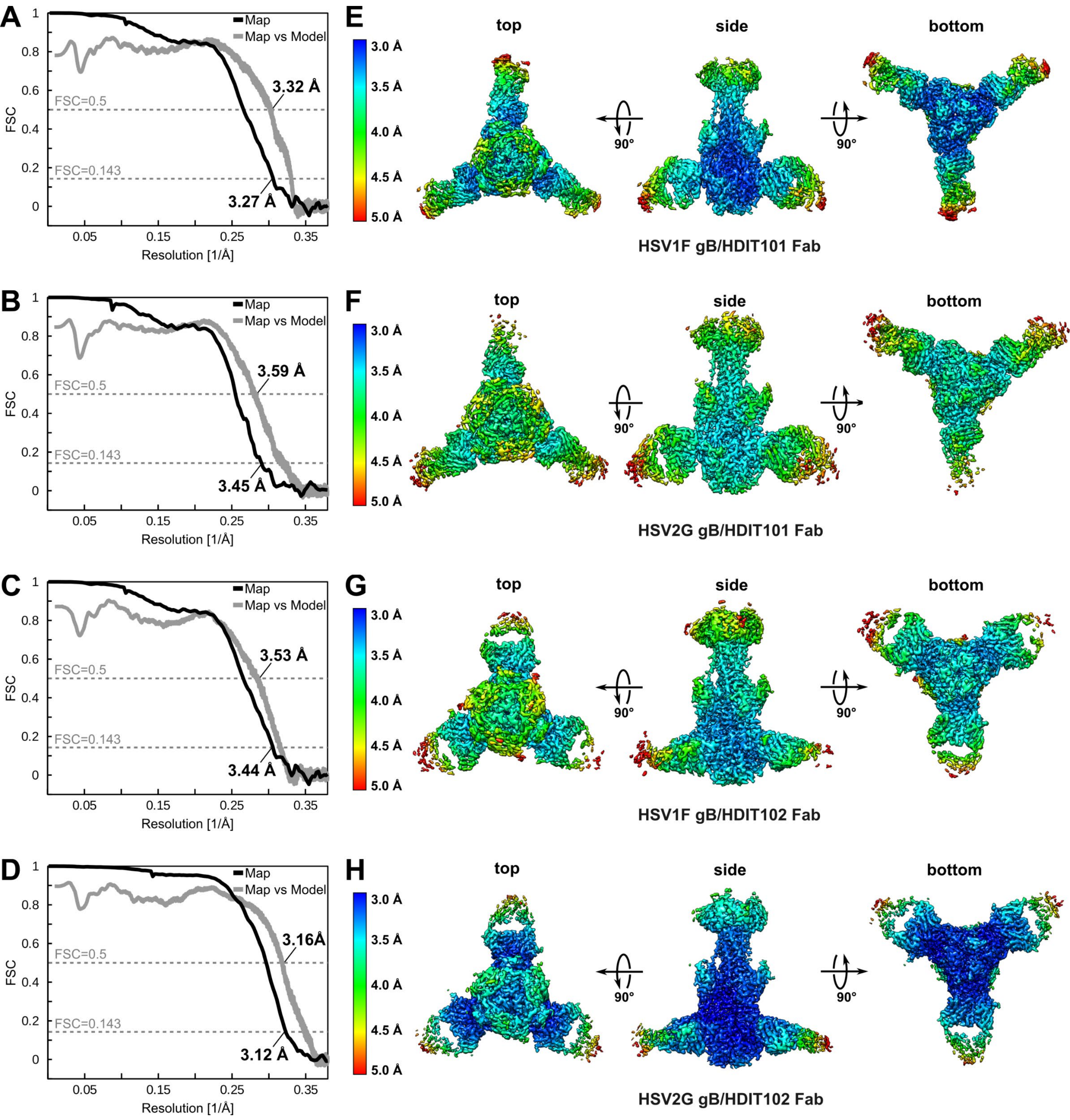
A
B
C
D
FSC
Resolution [1/Å]
Map
Map vs Model
FSC=0.5
FSC=0.143
3.32 Å
3.27 Å
3.59 Å
3.45 Å
3.53 Å
3.44 Å
3.16Å
3.12 Å
E
F
G
H
top
side
bottom
90°
3.0 Å
3.5 Å
4.0 Å
4.5 Å
5.0 Å
HSV1F gB/HDIT101 Fab
HSV2G gB/HDIT101 Fab
HSV1F gB/HDIT102 Fab
HSV2G gB/HDIT102 Fab

Figure S4

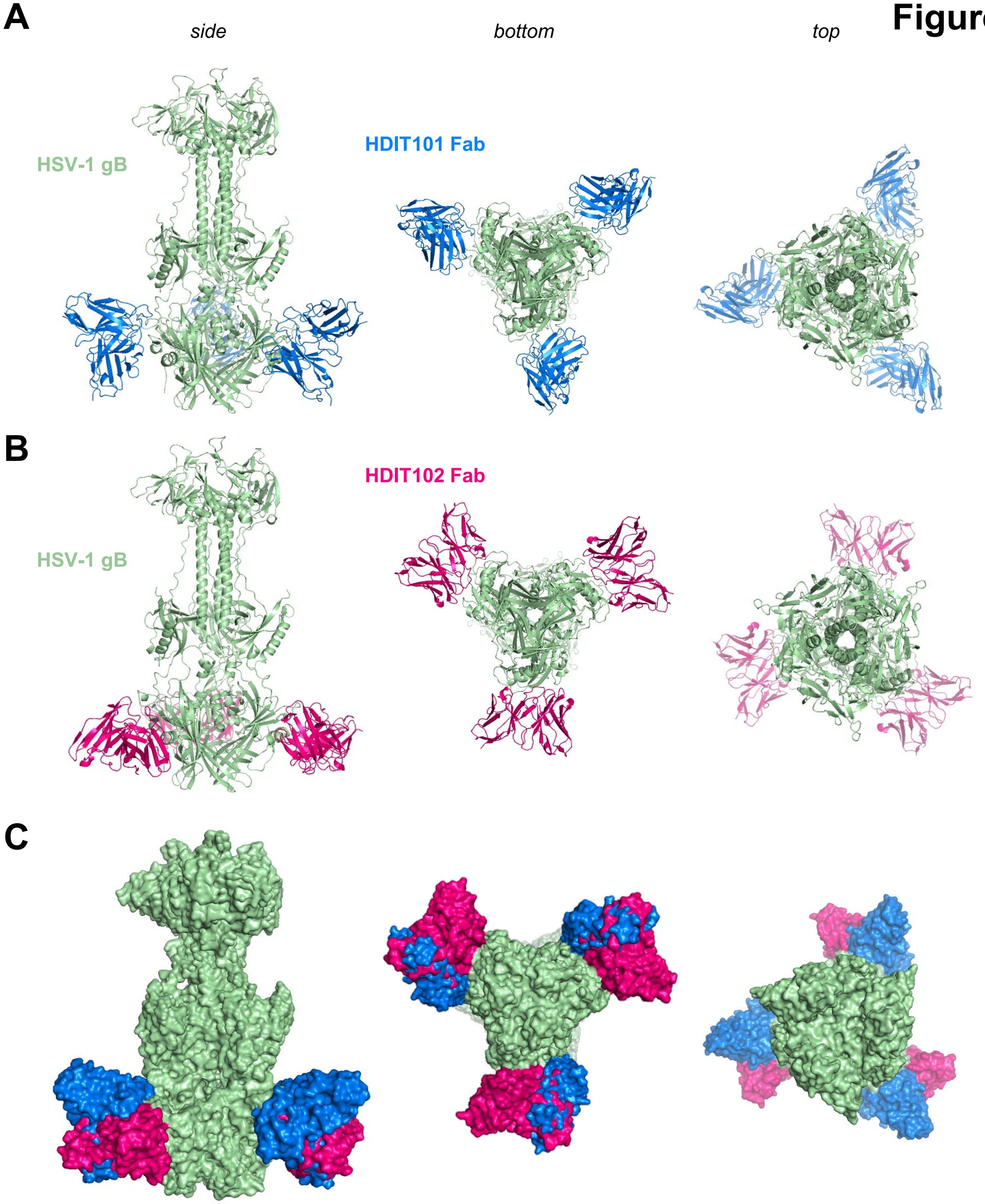

Figure S4

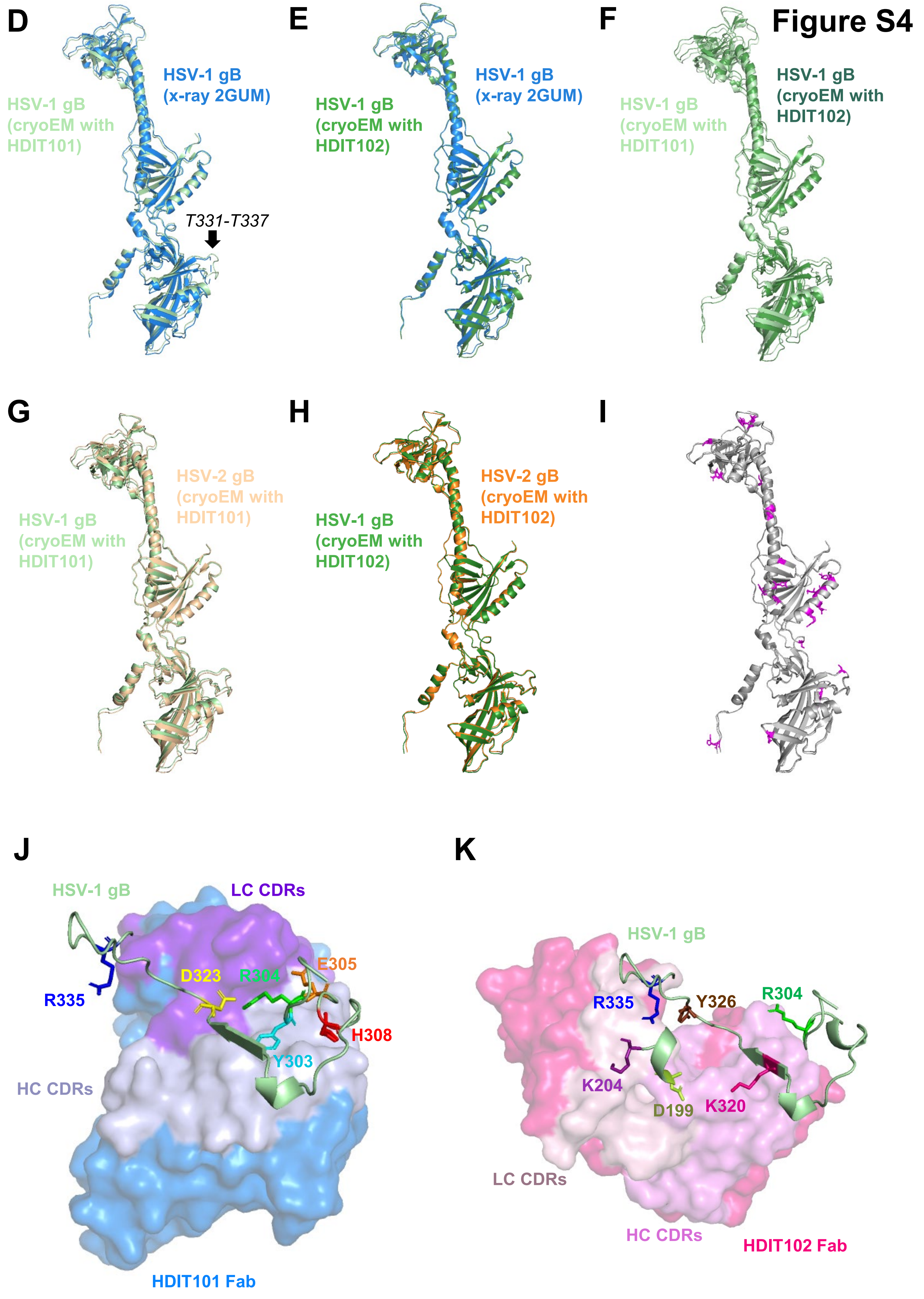

D
HSV-1 gB (cryoEM with HDIT101)
HSV-1 gB (x-ray 2GUM)
T331-T337
E
HSV-1 gB (cryoEM with HDIT102)
HSV-1 gB (x-ray 2GUM)
F
HSV-1 gB (cryoEM with HDIT101)
HSV-1 gB (cryoEM with HDIT102)
G
HSV-1 gB (cryoEM with HDIT101)
HSV-2 gB (cryoEM with HDIT101)
H
HSV-1 gB (cryoEM with HDIT102)
HSV-2 gB (cryoEM with HDIT102)
I
J
HSV-1 gB
LC CDRs
R335
D323
R304
E305
H308
Y303
HC CDRs
HDIT101 Fab
K
HSV-1 gB
R335
Y326
R304
K204
D199
K320
LC CDRs
HC CDRs
HDIT102 Fab

Figure S5

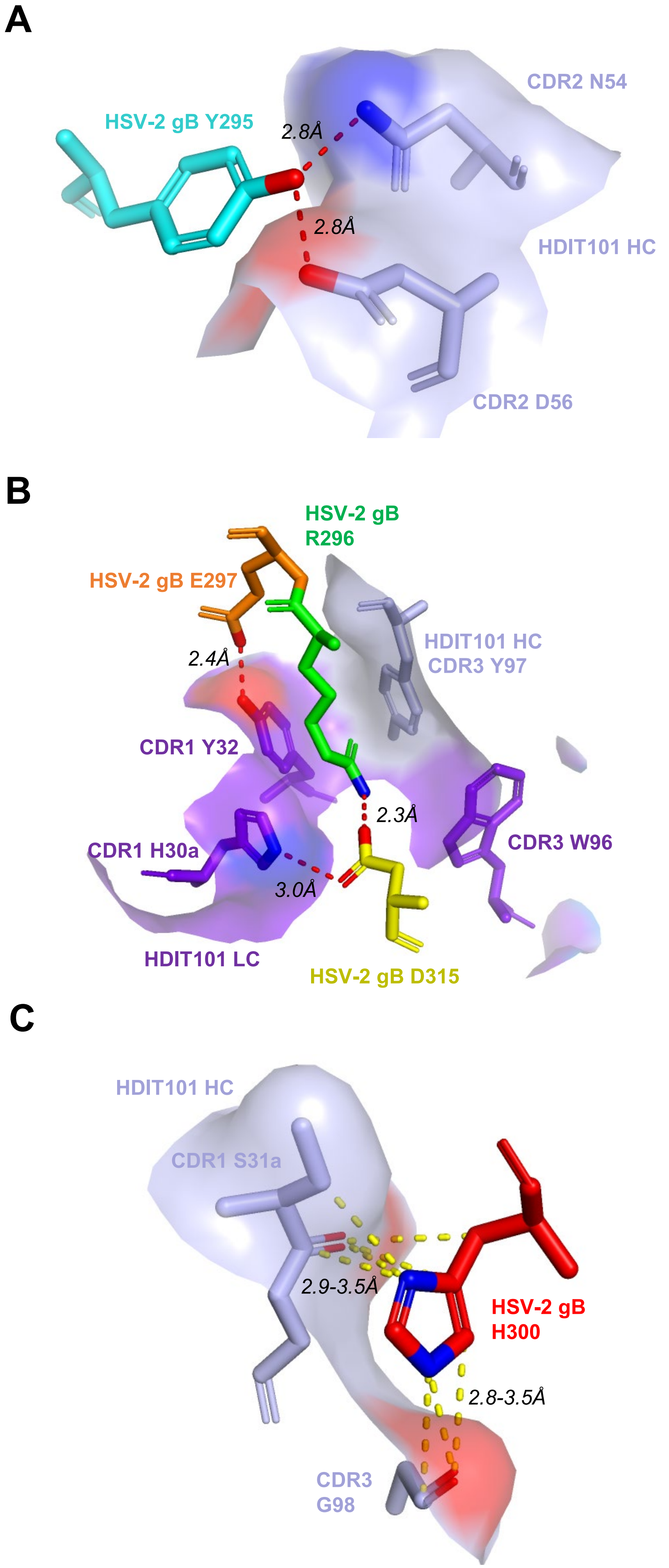

A
HSV-2 gB Y295
2.8Å
CDR2 N54
2.8Å
HDIT101 HC
CDR2 D56
B
HSV-2 gB
R296
HSV-2 gB E297
2.4Å
HDIT101 HC
CDR3 Y97
CDR1 Y32
2.3Å
CDR3 W96
CDR1 H30a
3.0Å
HDIT101 LC
HSV-2 gB D315
C
HDIT101 HC
CDR1 S31a
2.9-3.5Å
HSV-2 gB
H300
2.8-3.5Å
CDR3
G98

Figure S5

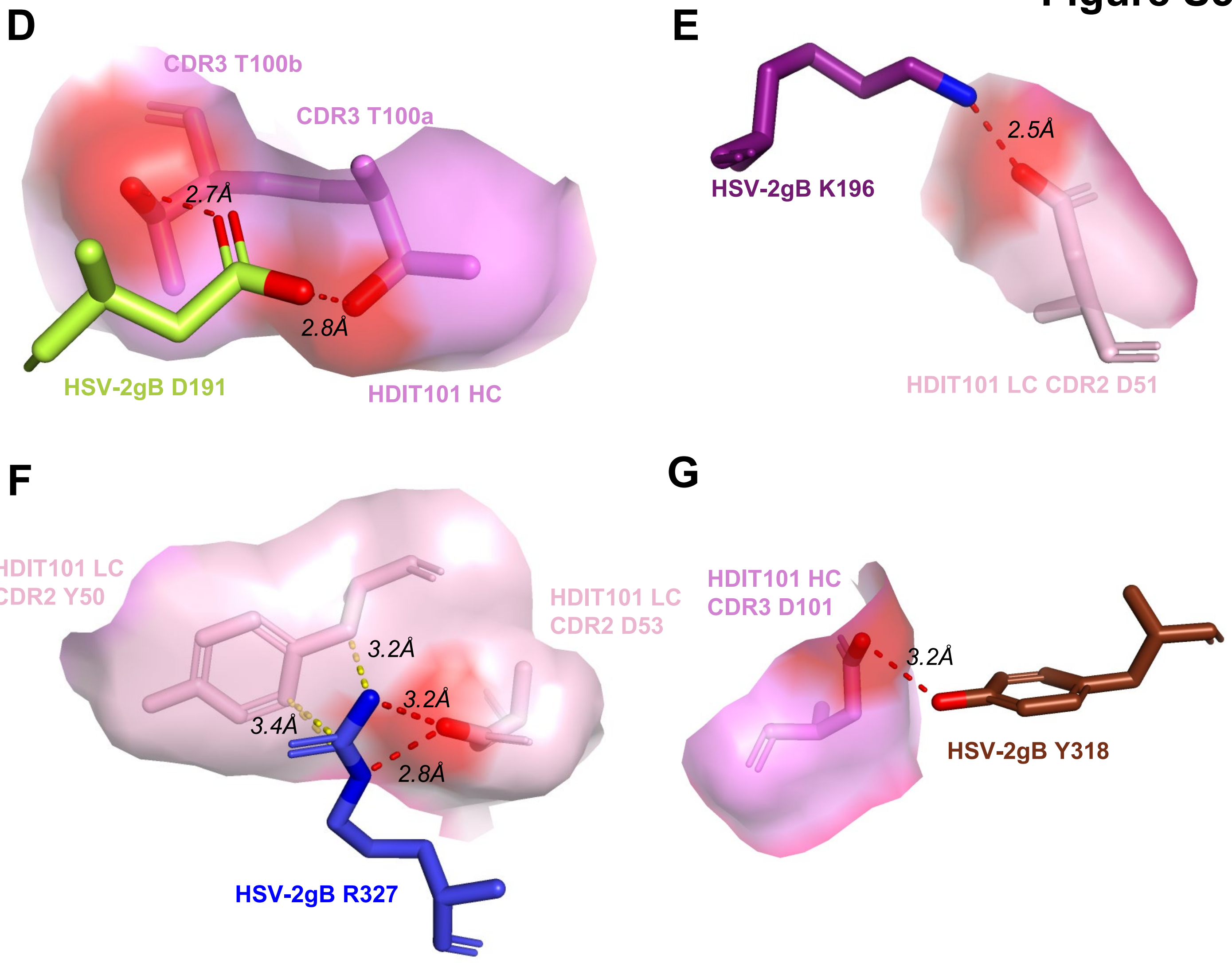
D
CDR3 T100b
CDR3 T100a
2.7Å
2.8Å
HSV-2gB D191
HDIT101 HC
E
HSV-2gB K196
2.5Å
HDIT101 LC CDR2 D51
F
HDIT101 LC
CDR2 Y50
HDIT101 LC
CDR2 D53
3.2Å
3.2Å
3.4Å
2.8Å
HSV-2gB R327
G
HDIT101 HC
CDR3 D101
3.2Å
HSV-2gB Y318

A **Figure S6**

```
HSV-1F gB    181 QFMGIFEDRAPVPFEEVIDKINAKGVCRSTAKYVRNNLETTAFHRDDHETDMELKPANAA 240
                 QFMGIFEDRAPVPFEEVIDKINAKGVCRSTAKYVRNN+ETTAFHRDDHETDMELKPA  A
HSV-2G gB    173 QFMGIFEDRAPVPFEEVIDKINAKGVCRSTAKYVRNNMETTAFHRDDHETDMELKPAKVA 232

HSV-1F gB    241 TRTSRGWHTTDLKYNPSRVEAFHRYGTTVNCIVEEVDARSVYPYDEFVLATGDFVYMSPF 300
                 TRTSRGWHTTDLKYNPSRVEAFHRYGTTVNCIVEEVDARSVYPYDEFVLATGDFVYMSPF
HSV-2G gB    233 TRTSRGWHTTDLKYNPSRVEAFHRYGTTVNCIVEEVDARSVYPYDEFVLATGDFVYMSPF 292
                    304                                    335
HSV-1F gB    301 YGYREGSHTEHTSYAADRFKQVDGFYARDLTTKARATAPTTRNLLTTPKFTVAWDWVPKR 360
                 YGYREGSHTEHTSYAADRFKQVDGFYARDLTTKARAT+PTTRNLLTTPKFTVAWDWVPKR
HSV-2G gB    293 YGYREGSHTEHTSYAADRFKQVDGFYARDLTTKARATSPTTRNLLTTPKFTVAWDWVPKR 352
                    296                                    327
HSV-1F gB    361 PSVCTMTKWQEVDEMLRSEYGGSFRFSSDAISTTFTTNLTEYPLSRVDLGDCIGKDARDA 420
                 P+VCTMTKWQEVDEMLR+EYGGSFRFSSDAISTTFTTNLT+Y LSRVDLGDCIG+DAR+A
HSV-2G gB    353 PAVCTMTKWQEVDEMLRAEYGGSFRFSSDAISTTFTTNLTQYSLSRVDLGDCIGRDAREA 412
```

Figure S6

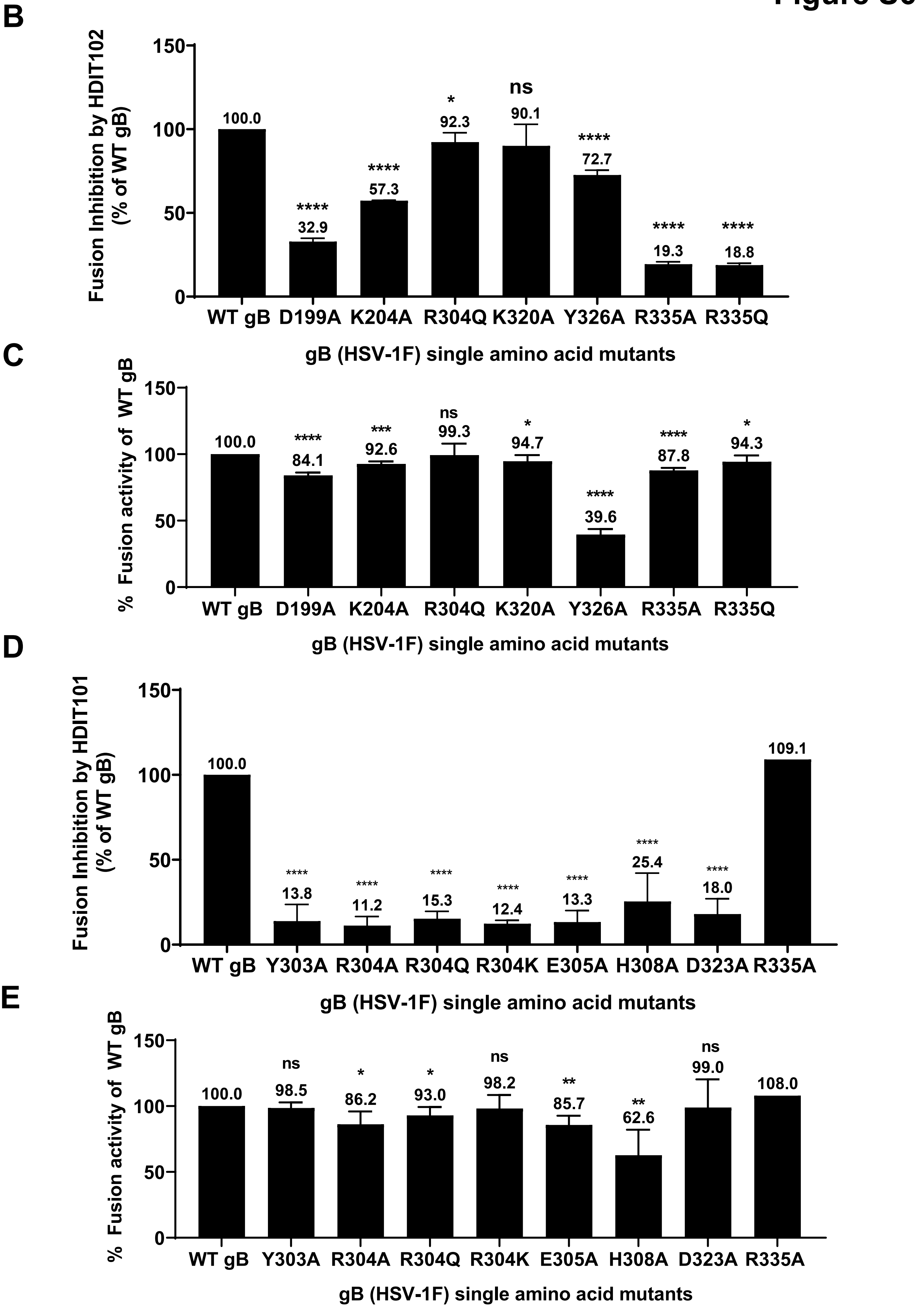
B
Fusion Inhibition by HDIT102 (% of WT gB)
150
100
50
0
100.0
****
32.9
****
57.3
*
92.3
ns
90.1
****
72.7
****
19.3
****
18.8
WT gB D199A K204A R304Q K320A Y326A R335A R335Q
gB (HSV-1F) single amino acid mutants
C
% Fusion activity of WT gB
150
100
50
0
100.0
****
84.1
***
92.6
ns
99.3
*
94.7
****
39.6
****
87.8
*
94.3
WT gB D199A K204A R304Q K320A Y326A R335A R335Q
gB (HSV-1F) single amino acid mutants
D
Fusion Inhibition by HDIT101 (% of WT gB)
150
100
50
0
100.0
****
13.8
****
11.2
****
15.3
****
12.4
****
13.3
****
25.4
****
18.0
109.1
WT gB Y303A R304A R304Q R304K E305A H308A D323A R335A
gB (HSV-1F) single amino acid mutants
E
% Fusion activity of WT gB
150
100
50
0
100.0
ns
98.5
*
86.2
*
93.0
ns
98.2
**
85.7
**
62.6
ns
99.0
108.0
WT gB Y303A R304A R304Q R304K E305A H308A D323A R335A
gB (HSV-1F) single amino acid mutants

Figure S6

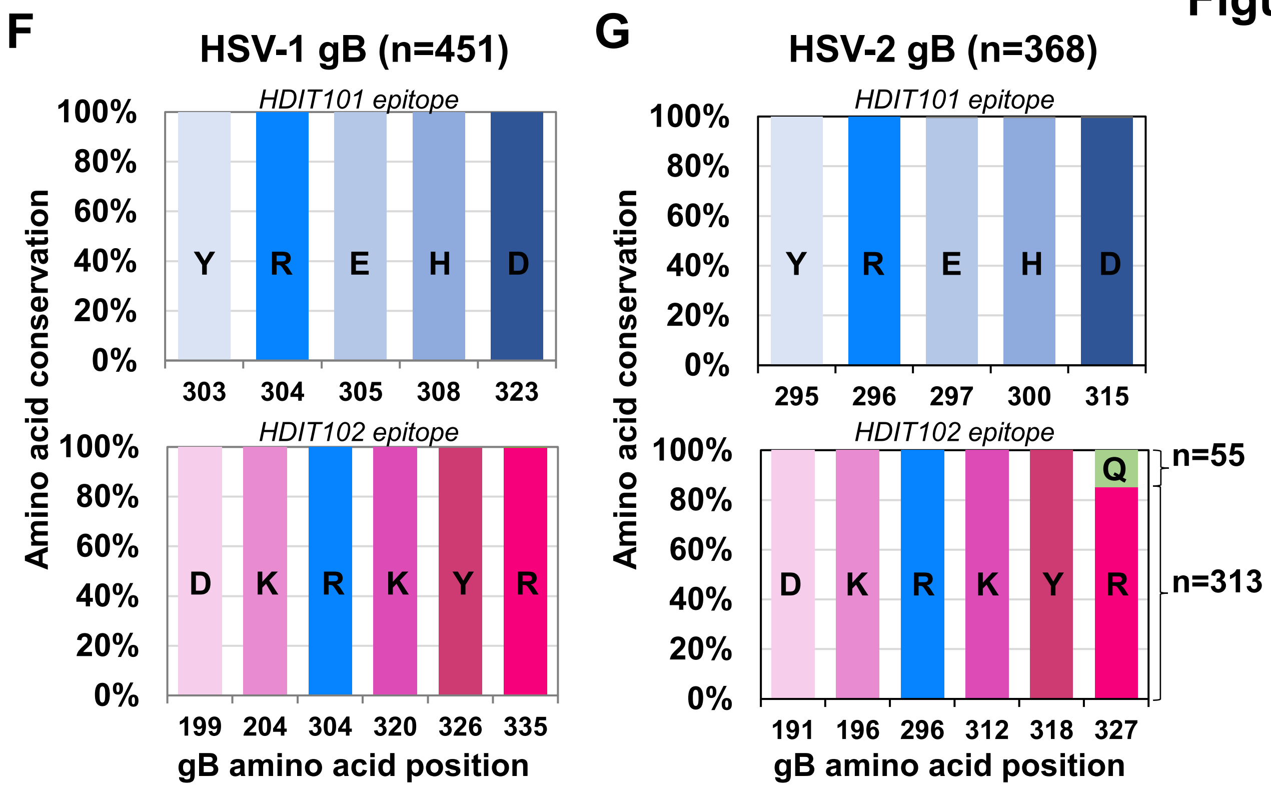

F
HSV-1 gB (n=451)
HDIT101 epitope
100%
80%
60%
40%
20%
0%
Y
R
E
H
D
303
304
305
308
323
HDIT102 epitope
D
K
R
K
Y
R
199
204
304
320
326
335
Amino acid conservation
gB amino acid position
G
HSV-2 gB (n=368)
HDIT101 epitope
Y
R
E
H
D
295
296
297
300
315
HDIT102 epitope
Q
D
K
R
K
Y
R
191
196
296
312
318
327
n=55
n=313
Amino acid conservation
gB amino acid position

Figure S6

H

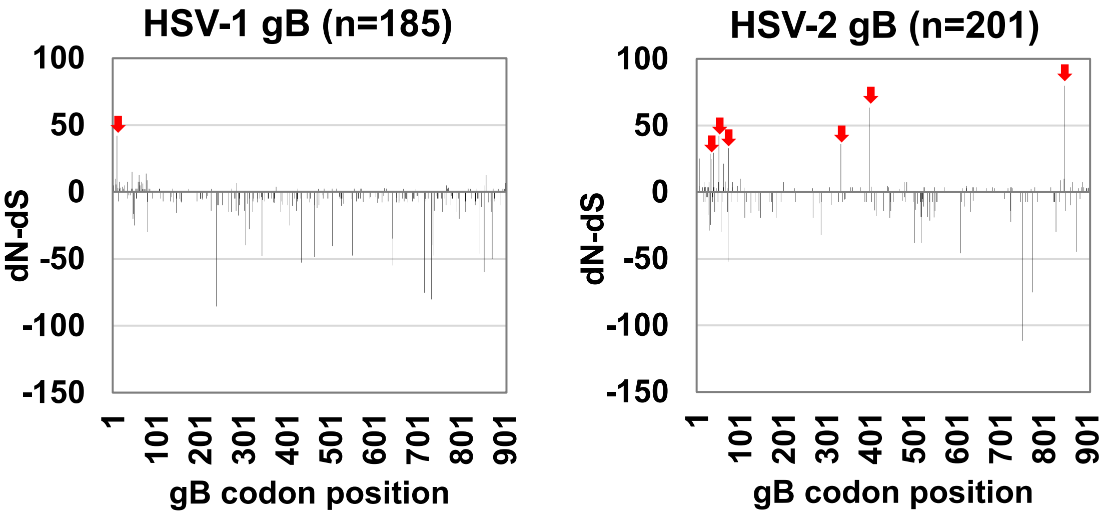
HSV-1 gB (n=185)
HSV-2 gB (n=201)
dN-dS
100
50
0
-50
-100
-150
1
101
201
301
401
501
601
701
801
901
gB codon position

I

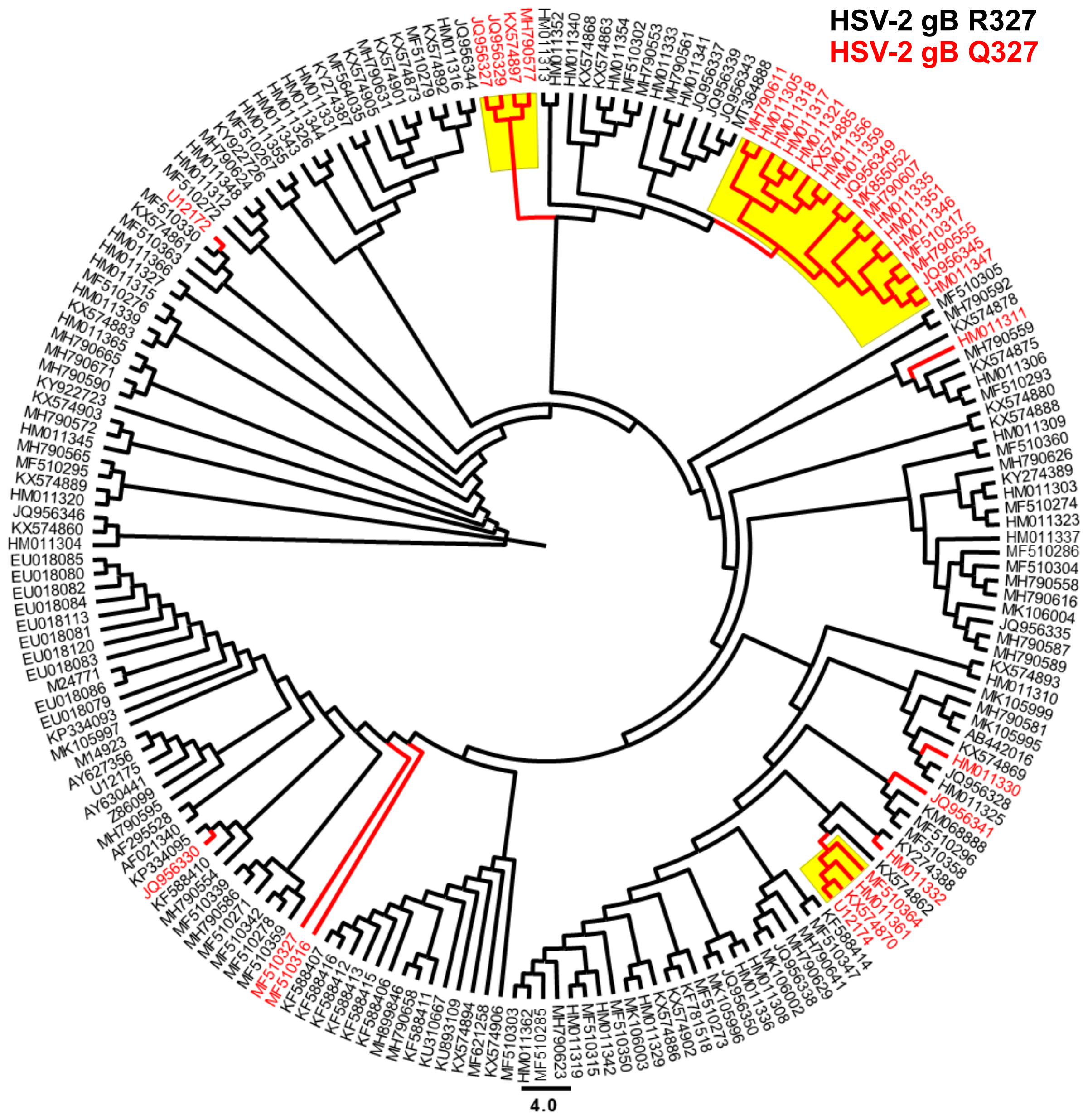
HSV-2 gB R327
HSV-2 gB Q327
4.0

Figure S7

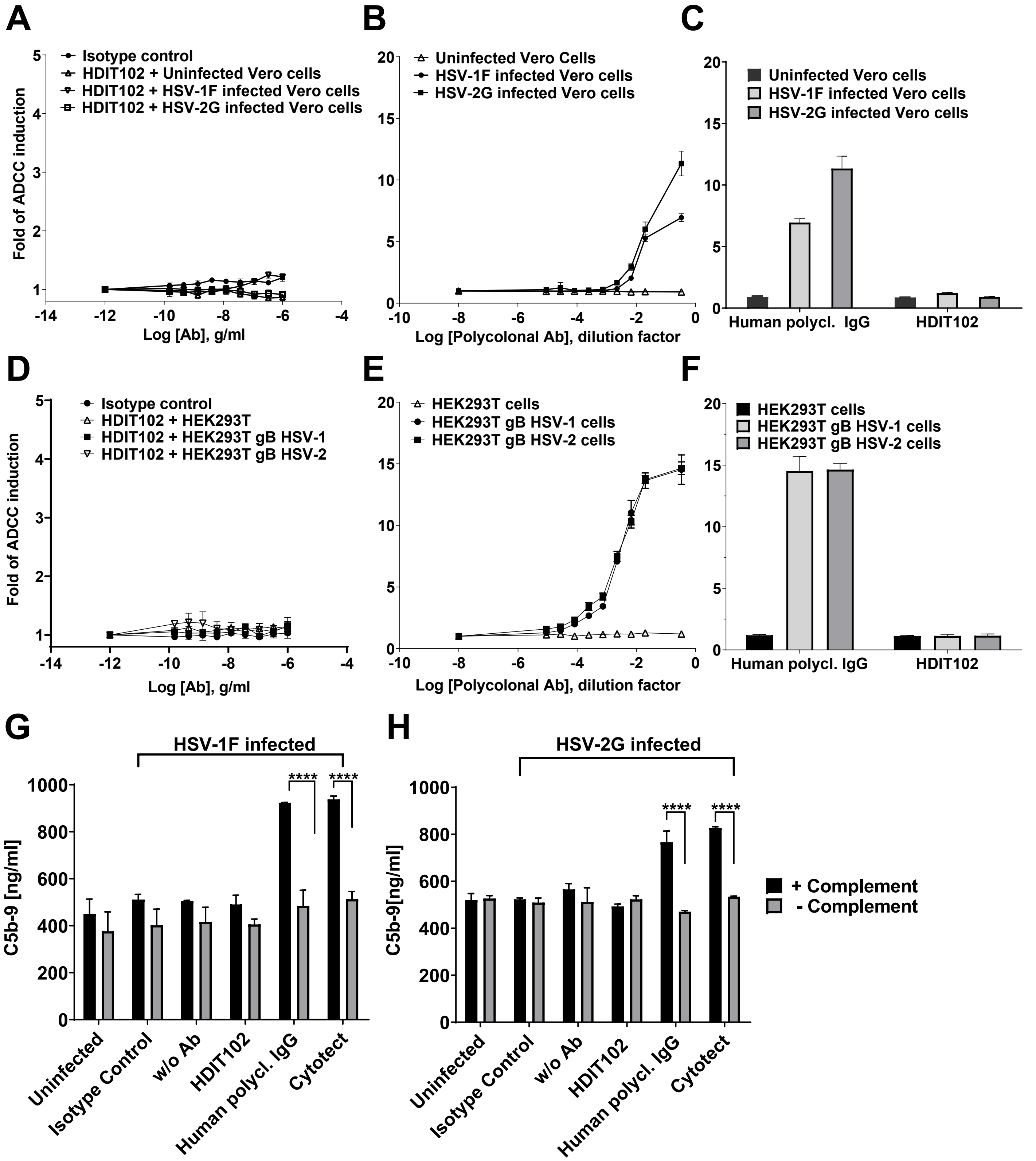

A
Isotype control
HDIT102 + Uninfected Vero cells
HDIT102 + HSV-1F infected Vero cells
HDIT102 + HSV-2G infected Vero cells
Fold of ADCC induction
Log [Ab], g/ml
B
Uninfected Vero Cells
HSV-1F infected Vero cells
HSV-2G infected Vero cells
Log [Polycolonal Ab], dilution factor
C
Uninfected Vero cells
HSV-1F infected Vero cells
HSV-2G infected Vero cells
Human polycl. IgG
HDIT102
D
Isotype control
HDIT102 + HEK293T
HDIT102 + HEK293T gB HSV-1
HDIT102 + HEK293T gB HSV-2
Fold of ADCC induction
Log [Ab], g/ml
E
HEK293T cells
HEK293T gB HSV-1 cells
HEK293T gB HSV-2 cells
Log [Polycolonal Ab], dilution factor
F
HEK293T cells
HEK293T gB HSV-1 cells
HEK293T gB HSV-2 cells
Human polycl. IgG
HDIT102
G
HSV-1F infected
C5b-9 [ng/ml]
****
****
Uninfected
Isotype Control
w/o Ab
HDIT102
Human polycl. IgG
Cytotect
H
HSV-2G infected
C5b-9[ng/ml]
****
****
Uninfected
Isotype Control
w/o Ab
HDIT102
Human polycl. IgG
Cytotect
+ Complement
- Complement
